# Transcriptomic shift in ethanol and amino acid metabolic genes regulated by Med15 during alcoholic fermentation

**DOI:** 10.1099/mgen.0.001811

**Published:** 2026-09-03

**Authors:** David G. Cooper, Emma Grunkemeyer, Jan S. Fassler

**Affiliations:** 1 Department of Biology, University of Iowa, Iowa City, IA, USA; 2 Department of Pharmaceutical Sciences, Butler University, Indianapolis, IN, USA

**Keywords:** arginine, fermentation, Med15, polyglutamine tracts, RNA-Seq, wine yeast

## Abstract

Organisms that thrive in extreme environments provide natural experiments in evolution, revealing the genetic regulators that orchestrate complex phenotypic change. Wine yeast (WY) are specialized strains that are adapted to survive in the wine making environment while producing high concentrations of ethanol. In addition to large genomic changes that differentiate WY from yeast used in other industries, SNP and polyglutamine tract polymorphism in the transcriptional regulator Med15 are associated with the fermentation efficiency and stress response phenotypes of WY. In this study, we investigated the transcriptional differences during wine fermentation in transgenic lab strain yeast having integrated WY *MED15* alleles. Compared to the unmodified lab strain (*MED15*_LAB_), the same strain in which the *MED15* locus was replaced with a *MED15* allele from yeast isolated from palm wine, the fermented sap of palm (oil, date, coconut) trees (*MED15*_WY23_), exhibited enhanced expression of amino acid biosynthesis genes as well as stress resistance and metabolic adaptation genes. Our experimental data confirm the role of arginine in efficient fermentation and suggest that certain *MED15* alleles alter the expression patterns of arginine pathway genes in some cases improving carbon flux under nitrogen stress. The global benefits conferred by natural polymorphisms in a single transcriptional regulator highlight Med15 as a target for engineering of strains devoted to various types of alcohol production.

**Impact Statement**

This study identifies polymorphisms in the transcriptional regulator Med15 as one key modulator of fermentation efficiency in wine yeast (WY). By integrating WY *MED15* alleles into laboratory strains, we demonstrate changes in expression of genes involved in glycolysis, fermentation and amino acid biosynthesis – particularly those in the arginine biosynthetic pathway. These findings provide mechanistic insight into how single-gene variation can drive complex phenotypic traits in industrial fermentation. The work highlights Med15 as a promising genetic target for engineering yeast strains optimized for diverse alcohol production environments, offering broad applications in biotechnology and microbial genomics and validates this approach for identifying broad drivers of evolutionary change.

## Data Summary

All Illumina RNA-Seq datasets are available in the National Centre for Biotechnology Information under BioProject accession PRJNA1320546. Data analysis is summarized in a Microbiology Society Figshare submission at https://doi.org/10.6084/m9.figshare.32791668 and in a GitHub repository with persistent link https://doi.org/10.5281/zenodo.17047964.

## Introduction

The production of wine requires yeast strains that can efficiently convert sugars to ethanol while also surviving the stresses of the grape juice/must environment including high osmolarity, low pH, low nitrogen availability and ultimately high ethanol concentrations. The general transcriptional regulator Med15 influences both fermentation efficiency and stress response during grape juice/must fermentation [1], and thus the *MED15* gene is a good candidate for domestication niche-specific adaptive mutations leading to improved fermentation observed in wine yeast (WY) [1, 2]. Variations in alleles of the general transcriptional regulator *MED15*, including the length of several polyglutamine (poly-Q) tracts embedded in Med15 sequence, are associated with yeast domestication niches (beer and wine) [2].

Although various yeast species are naturally present on grapes, *Saccharomyces cerevisiae* dominates the fermentation environment [3]. Historically, and as a modern trend, uninoculated ‘natural fermentations’ have been used in the production of wine, although many wineries will specifically inoculate grape must with previously isolated and commercially available WY strains [4].

Fermentation reactions are characterized by an initial lag phase for yeast not pre-adapted to juice followed by a growth phase after which cells reach saturation (10^8^ cells ml^−1^). Available nitrogen and ~50% of the sugars are quickly consumed during the growth phase [5]. The remaining sugar is fermented after culture saturation. During this time, the sugar is used almost exclusively to generate energy and survival compounds rather than biomass [5]. Ethanol is the main byproduct of yeast fermenting glucose for energy. Other metabolic products are made throughout the fermentation process including various acids (acetic, succinic and lactic) and volatile compounds (esters, fatty acids, aldehydes and higher alcohols) [6–8]. Industrial fermentation reactions can be reliably replicated in the lab using small-scale fermentation reactions [1, 8].

In addition to environmental stresses such as high-sugar content osmotic stress, nitrogen limitation and low pH present at the start of fermentation, accumulation of both ethanol and acetic acid are stressors specific to late fermentation cultures [3]. Correspondingly, WY exhibit high tolerance to many compounds and stressors including acetic acid, copper, sulfite, high glucose, sorbitol and ethanol [9]. Yeast respond to the initial and accumulating stresses of grape juice and fermentation through the environmental stress response (ESR) [10, 11]. The ESR allows yeast to deal with fermentation stress and moderate levels of ethanol but eventually the levels of alcohol become toxic. Ethanol toxicity is thought to occur by disrupting membrane structural integrity, while the addition of lipids or oxygenation, that is required to synthesize fatty acids, can mitigate the toxic effects [6, 7].

The *S. cerevisiae* yeast strains used in food and beverage production (e.g. wine, beer and bread) are phenotypically distinct from wild yeast strains and represent lineages from a small set of common ancestors [9, 12]. Prior to the isolation of specific WY strains, which are now removed from evolutionary processes by being maintained as stocks and sold to winemakers to add to grape must [13], WY diverged from wild yeast populations in numerous domestication events [4, 9, 14].

Across different alcoholic beverage industrial niches, *S. cerevisiae* yeast are critical for their ability to efficiently ferment various carbon sources to produce ethanol and carbon dioxide. Yeast will preferentially undergo alcoholic fermentation and accumulate ethanol even if oxygen is readily available for respiration [3], a phenomenon known as the Crabtree effect [15, 16]. After glycolysis, pyruvate is metabolized into acetaldehyde by pyruvate decarboxylases (PDCs) and acetaldehyde is converted into ethanol by alcohol dehydrogenases (ADHs). This step serves to restore NAD^+^ by oxidation of NADH that is produced during glycolysis. While ancestral ADH genes were optimized to produce ethanol, the whole genome duplication within the clade that includes *S. cerevisiae* resulted in ADH genes encoding enzymes with the ability to more efficiently consume ethanol instead of exclusively producing ethanol [3]. Specifically, Adh2 preferentially consumes ethanol with a Km for ethanol (600–800 µmol l^−1^) that is significantly lower than that for Adh1 (17,000–20,000 µmol l^−1^) [17, 18]. Thus, Adh2 works much more efficiently than Adh1 at oxidizing ethanol, while the remaining four Adh proteins (mitochondrial Adh3 and Adh4, cytoplasmic Adh1 and Adh5) preferentially produce ethanol [17]. Acetaldehyde produced by Adh2 is metabolized into acetate by aldehyde dehydrogenases (ALDs) and acetate is metabolized into acetyl CoA by acetyl CoA synthetases (ACSs) to be used in the citric acid cycle or other metabolic pathways.

The loss of *cis*-regulatory signals for respiration genes in yeast following the whole-genome duplication may have contributed to the Crabtree effect [19]. Specifically, yeast diverging after the whole-genome duplication, including *S. cerevisiae*, lost regulatory signals that would promote the expression of mitochondrial genes causing these yeast to display faster anaerobic growth compared to aerobic growth [19]. The ability of *S. cerevisiae* to preferentially ferment sugars and to tolerate higher levels of ethanol was likely selected for, as these traits allow *S. cerevisiae* to outcompete other yeast species in natural fermentation environments [3].

Med15 is a component of the tail module of the RNA polymerase II mediator complex [20–23]. As a general transcriptional regulator, Med15 has global effects on stress responses and metabolism [2]. Changes in the sequence of Med15 that influence activity could thus produce broad phenotypic changes, which provide the opportunity for yeast to adapt to novel environments. Variation in *MED15* sequence across strains predominantly consists of small changes in the length of simple sequence repeats that underlie glutamine and glutamine-alanine tracts. Recently, the phenotypic stress response consequences of Med15 poly-Q variation have been investigated [1, 24, 25]. Specifically, Med15 was found to be required for resistance to the coal-cleaning chemical, 4-methylcyclohexane methanol (MCHM) and changes in poly-Q tract lengths were found in yeast resistant to MCHM relative to the endogenous *MED15* allele in the lab strain [24]. Our previous work revealed that some of the fermentation efficiency characteristic of WY strains is conferred by SNPs and poly-Q tract length polymorphisms in Med15 [1]. We have found that the disorder promoted by glutamine residues is important for Med15 function and that variation in poly-Q tract length in Med15 can modulate stress response and metabolic activity [25]. The significance of poly-Q tract length variation has also been investigated in the yeast Cyc8 transcriptional co-repressor protein [26], as well as in the ANGUSTIFOLIA and Clock proteins in other organisms, where even small changes in tract length (two to four additional glutamine residues) are associated with noticeable phenotypic changes [27–29].

Here we conduct, to our knowledge, one of the first analyses of the *MED15* transcriptome during white grape juice (WGJ) fermentation to identify *MED15*-regulated genes that are unique to the fermentation environment compared to standard lab conditions. In addition, we test the hypothesis that the *MED15* alleles from WY harbouring variable length poly-Q tracts and other dispersed SNPs [1, 2] may have contributed to specialized fermentation transcriptomes by comparing the fermentation transcriptome of lab strains into which we have introduced WY *MED15* alleles (labelled *MED15*_WY##_) to the unmodified lab strain (*MED15*_LAB_) background. Briefly, our results show that *MED15* regulates glycolytic and fermentation pathways, with a particularly strong and distinctive regulatory effect during fermentation and that some *MED15_WY_* alleles cause subtle but biologically significant changes in metabolism when substituted for the *MED15* locus in the lab strain. We report the transcriptomic basis for the improvement in fermentation rates seen in specific alcoholic beverage yeasts previously characterized [1], and although we expected changes to genes involved in glycolysis and ethanol metabolism due to the *MED15*_WY_ alleles, we instead observed differences in the expression of amino acid metabolic genes, among others, compared to unmodified *MED15*_LAB_ strains. These findings suggest that multiple distinct transcriptomic profiles can enhance fermentation rates.

## Methods

### Strains and plasmids

Strains used in this study are derivatives of S288C, a widely used non-flocculent laboratory strain, originally designed for biochemical studies [30]. The *med15* deletion strain is from the S288C *MAT*α haploid (BY4742) deletion collection [31]. Genotypes of additional strains used in this study are listed in Table 1. WY strains were obtained from the Agricultural Research Service (ARS) culture collection (https://nrrl.ncaur.usda.gov/) (WY23, palm WY, NRRL-Y17772) or were from the Goddard laboratory collection [32] (WY7, Lalvin ICV D-254; WY15, DSM Fermichamp). Plasmids used in this study are listed in Table 2.

**Table 1.** Strains

Strain*	Relevant genotype	Parental strain and /or plasmid	Source or reference
BY4742	MATα *his3Δ1 leu2Δ0 lys2Δ0 ura3Δ0*	(OY235)	[31]
(OY320†)	*med15Δ0*::KanMX4	BY4742 deletion collection	[31]
JF2711	*med15Δ0*::*URA3*	BY4742 with a *URA3*-marked *MED15* deletion locus	This study
JF2692	*MED15*_LAB_	pJF2141 (episomal) in OY320	This study
JF2641	*MED15*_WY7_	pJF2155 (episomal) in OY320	This study
JF2642	*MED15*_WY15_	pJF2156 (episomal) in OY320	This study
JF2646	*MED15*_WY23_	pJF2158 (episomal) in OY320	This study
JF2754	*MED15*_LAB_	*MED15* ORF from pJF2141 integrated at *MED15* locus in JF2711	This study
JF2756	*MED15*_WY7_	*MED15* ORF from pJF2155 integrated at *MED15* locus in JF2711	This study
JF2758	*MED15*_WY15_	*MED15* ORF from pJF2156 integrated at *MED15* locus in JF2711	This study
JF2760	*MED15*_WY23_	*MED15* ORF from pJF2158 integrated at *MED15* locus in JF2711	This study
WY7	Diploid WY	–	Lalvin ICV D254
WY15	Diploid WY	–	DSM Fermichamp
WY23	Diploid WY	–	NRRL Y-17772

* All strains except for those designated WY carry the *ura3Δ0* mutation. JF2711 carries the same mutation but is Ura+due to the *URA3*+ marker at the *MED15* locus.

† Parenthetical names (OY###) are lab-specific storage designations.

**Table 2.** Plasmids

Plasmid	Detail	Other comment	Source or reference
pRS315	*LEU2*, CEN	(SV31)	[96]
YEp24	*URA3*, 2μ	(SV6)	–
pRS315 M-WT	Mini-*MED15;* the internal region of Med15 (aa 116–277) plus the Mediator association domain (aa 799–1,081) located between the promoter and terminator regions of S288C *MED15* in pRS315 (*LEU2*, CEN)	(SV286)	[33]
pJF2141	BY4742 *MED1*5 in pRS315 (*LEU2*, CEN)	SV286 gap repair	This study
pJF2155	*MED15*_WY7_ in pRS315 (*LEU2*, CEN)	SV286 gap repair	This study
pJF2156	*MED15*_WY15_ in pRS315 (*LEU2*, CEN)	SV286 gap repair	This study
pJF2158	*MED15*_WY23_ in pRS315 (*LEU2*, CEN)	SV286 gap repair	This study

Parenthetical names (SV###) are lab storage designations.

### Wine yeast *MED15* integration

A *URA3*-marked *med15Δ* lab strain (JF2711) was created to use as a starting point for integrating WY *MED15* alleles. A *URA3* expression cassette was amplified from YEp24 using *URA3* GR (gap repair) F1 and R1 primers (Table 3). Primers were complementary to sequences outside of the *MED15* ORF, allowing for homology to be used in the gap repair of *Bam*HI- and *Spe*I-digested Mini-*MED15* plasmid (pRS315 M-WT) [33]. The *URA3* expression cassette with *MED15* flanking homology was subsequently amplified from the newly constructed plasmid using *MED15* F-245 and *MED15* R+3498 primers (Table 3) and integrated into the genome of BY4742 at the *MED15* locus to generate JF2711. WY *MED15* alleles were integrated into the *MED15* locus of JF2711 by homologous recombination. *MED15* coding sequences with 250 bp of upstream and downstream sequence were amplified from expression plasmids (pJF2141, LAB; pJF2155, WY7; pJF2156, WY15; pJF2158, WY23) using *MED15* F-245 and *MED15* R+3498 primers (Table 3). *MED15* PCR fragments were then transformed into JF2711 and cells plated on yeast peptone dextrose (YPD) medium. After a day of growth, plates containing 800 mg l^−1^ 5-fluoroorotic acid (5-FOA) were used to counter-select transformants that had become Ura- due to integration of the new allele and loss of the *URA3*-marked *MED15* locus. Integrants were stored as JF2754-JF2760.

**Table 3.** Primers

Primer	Sequence (5′–3′)	Use
*MED15* F-245	GGATGAGGATGATGAAGGTGC	Amplify *MED15* ORF
*MED15* R+3498	GTACTGATGATAGTCAAGTCCATTG	Amplify *MED15* ORF
*MED15* F+384	GTTCTTGAATCAGCAGGCTC	Screen WY alleles
*MED15* R+529	GTGCCACTTTCATCTGGTTC	Screen and sequencing
*MED15* F+1924	GGCACTACTTCTACTGGAAAC	Screen WY alleles
*MED15* R+2143	CATTAGCCATTGCCGAATAATTAGCCACACCAGG	Screen WY alleles
*MED15* F+2693	AGATGCTTACGTCATGCACTATCC	Sequencing
*MED15* R+2990	GGGTTGCCGACATCCATATTAG	Sequencing
*URA3* GR F1	TCGTATAGTGCCGTACTCAAAGATCAAGGATTAAAACGCGACAGCTTATCATCGATAAGC	Amplify *URA3* gene
*URA3* GR R1	GTATCAAAAGTATGGAAACTTCAAATGTTCAAGTAGCACAATCATTACGACCGAGATTCC	Amplify *URA3* gene

### Colony PCR

Yeast colonies were used for rapid PCR screening using Taq polymerase, colony PCR buffer (final concentrations: 12.5 mM Tris/Cl (pH 8.5), 56 mM KCl, 1.5 mM MgCl2, 0.2 mM deoxynucleotide triphosphates (dNTPs)) and primers at a final concentration of 0.2 µM. Reaction mixes were aliquoted into PCR tubes into which a small amount of yeast cells were transferred using the end of a 200 µl micropipette tip. A standard hot-start thermocycler programme with extension at 68 °C was adjusted for the Tm of the primer set and size of the amplification product.

### Yeast transformation

Transformations of *med15Δ* strains were conducted using the Frozen-EZ Yeast Transformation II kit (Zymo Research) with modifications. A total of 10 ml of early log phase cells were pelleted and washed with 2 ml EZ 1 solution, repelleted and resuspended in 1 ml EZ 2 solution. Aliquots were frozen at −80 °C. A total of 0.2–1 µg DNA and 100 µl of EZ 3 solution were added to 10 µl competent cells for each transformation. Transformations were plated on selective media following incubation at 30 °C for 45 min. Transformations into all other strains were conducted using a standard lithium acetate transformation protocol [34, 35].

### Small-scale fermentation reactions

WGJ concentrate (Global Vinters Inc., Canada) was supplied at ~68°Bx (1°Bx is equal to 0.01 g ml^–1^) and was diluted to 20–21°Bx with distilled deionized water as measured using a triple scale hydrometer and confirmed with a refractometer (Milwaukee). Grape juice media was supplemented with 200 mg l^−1^ histidine, 300 mg l^−1^ leucine, 300 mg l^−1^ lysine and 200 mg l^−1^ uracil for use in growth and fermentation experiments.

Ten millilitre moulded sterile clear glass vials (American Clinical Supplies Inc.) were used in fermentation experiments. A total of 5 ml of prepared grape must (or other specified media) was added to each vial which was then closed tightly with a rubber cap. A 25 G × 7/8 inch (0.5×22 mm) hypodermic needle was inserted into the cap to allow CO_2_ release from the system and to restrict evaporation [8]. Prior to inoculating the fermentation vials, cells were grown in SC-Leu media for 2.5 days to reach saturation and then diluted 1 : 20 into fresh SC-Leu media. 1.25×10^7^ cells of saturated culture was added to 5 ml prepared white grape must (or other media as indicated in the figure legends) to a final cell concentration of 2.5×10^6^ cells ml^−1^. SC-Leu media was added as needed to standardize the media added to each vial. Uninoculated vials with the same volume of grape must were used to control for contamination. Fermentation vials were incubated at 25±1 °C or as indicated. The temperature was maintained at 25 °C despite fluctuating room temperatures (RTs) using a heated shaker in a 4 °C cold room. Shaker speed was maintained at 165 r.p.m.

In later fermentation experiments, synthetic complete media (SC) was used rather than WGJ. SC-arginine was 20% glucose and contained all amino acids except arginine.

### Fermentation assays

Progression through the fermentation process was determined by once-daily weight loss measurements caused by CO_2_ release. Cumulative CO_2_ weight loss was calculated by subtracting daily measurements from the initial weight (day 0). To examine the impact of each *MED15* plasmid, fermentation (weight loss) was evaluated simultaneously for multiple different transformants (biological replicates). Time to weight loss benchmarks were determined per transformant. The total amount of CO_2_ weight loss accumulated by day 7 for *MED15* strains or day 8 for *med15Δ* strains was set as 100% weight loss. Values equal to 10, 35, 50 and 80% of the 100% value were plotted as horizontal lines on top of fermentation curves. The time, in hours, that corresponded to the intersection between the horizontal lines and fermentation curves was determined manually or by linear interpolation.

### Analysis of microarray datasets

Microarray datasets used in the comparisons shown in Figs 3a and 5 were restricted to those that evaluated *med15*Δ and wild-type S288C strains after growth to mid log phase in media containing 2% glucose [36, 37]. The *med15*Δ strain in Hu *et al*. [36], reanalysed in Reimand *et al*. [38], was from the Open BioSystems collection in the BY4741 strain background. Cells were grown in YPD (2% glucose) and harvested at mid-log; the *med15*Δ strain was harvested at an OD_600_ of 0.88. Unaltered *med15*Δ data (log2 fold-change and*P*-values) were retrieved from provided processed data tables (E-MTAB-109 from ArrayExpress).

The *med15Δ* strain in Kemmeren *et al*. [37] was constructed by the gene deletion consortium in the MATα haploid BY4742 strain [39] and was harvested in log phase growth in SC media (2% glucose) in which the authors report the wild-type strain has the same doubling time as in YPD. Cultures were harvested at an OD_600_ of 0.6. Unaltered *med15Δ* data [log2 fold-change (m-values) and *P*-values] was downloaded from provided supplementary files (GSE42527 from Gene Expression Omnibus).

Genes with provided expression values surpassing a threshold log2 fold-change of >1 or <−1 and a *P*-value<0.05 were assembled into differentially regulated gene (DRG) sets that were used in the comparisons shown in Fig. 3a. All subtelomeric genes from the *COS*, *FLO*, *MAL* and *PAU* gene families, regardless of log2 fold-change and *P*-value cutoffs, were compiled as shown in Fig. 5.

### RNA extraction

RNA was extracted from 10 ml yeast cultures harvested in log phase (2×10^7^ cells ml^−1^). Cells were pelleted by centrifugation and resuspended in 1 ml cold water. Yeast were repelleted by centrifuging for 10 s at highest setting of tabletop centrifuge. The supernatant was aspirated, and the pellet was frozen in dry ice for 5 min before being stored at −80 °C prior to RNA extraction.

RNA was extracted using a hot acid phenol protocol [40]. Frozen cell pellets were resuspended in 400 µl TES solution (10 mM Tris, 10 mM EDTA, 0.5% SDS). An equal volume of acid phenol was added to each tube and vortexed for 10 s. Tubes were incubated in a 65 °C water bath for 1 h with vortexing every 10–12 min. Phases were separated by microcentrifugation in the cold for 5 min. The aqueous layer was extracted into a new tube avoiding the DNA-enriched interface. A second round of acid phenol extraction was conducted followed by a chloroform extraction. RNA was precipitated with 300 µl of 4 M LiCl in dry ice for 20 min followed by 5 min of high-speed centrifugation at 4 °C. The pellet was washed with 500 µl ice-cold 70% ethanol and dried for 10 min at 37 °C. RNA pellets were resuspended in DEPC-treated water and stored at −20 °C. Contaminating genomic DNA was removed using the DNase Max Kit (Qiagen). 10 µl of a 1x Master Mix (5 µl 10X buffer, 5 µl water and 0.5 µl DNase I) was added to 40 µl RNA. Following a 30 min incubation at 37 °C, DNase was removed by addition of 5 µl of the DNase removal resin and a 10 min RT incubation with periodic agitation. The resin was pelleted by centrifugation for 1 min at the highest setting of a microcentrifuge. The supernatant containing DNA-free RNA was transferred to a new tube, and the concentration was determined using a NanoDrop spectrophotometer (Thermo Scientific). RNA quality was visualized on formaldehyde gels (1% agarose, 10% formaldehyde solution, 1x MOPS buffer).

### cDNA preparation and quantitative reverse transcription polmerase chain reaction (qRT-PCR)

cDNA was prepared using Superscript III First-Strand Synthesis System (Invitrogen). Transcripts were amplified with random hexamer (Invitrogen) or anchored oligo-dT20 (Integrated DNA Technologies) primers. For anchored oligo-dT20 primers, 1 µg RNA, 50 ng primer and 0.01 µmol dNTPs were mixed in a final volume of 10 µl. Tubes were incubated for 5 min at 65 °C and then 2 min on ice. A total of 10 µl cDNA Master Mix (2 x RT buffer, 10 mM MgCl_2_, 0.02 M DTT, 40 U RNase OUT, 200 U Superscript III Reverse Transcriptase) or mock Master Mix lacking reverse transcriptase was added to each tube and incubated for 60 min at 50 °C and 5 min at 85 °C. To degrade RNA, 2 U RNase H was added to each tube and incubated for 20 min at 37 °C. For random hexamer primers, tubes were incubated 10 min at 25 °C and then 50 min at 50 °C.

Transcript abundance was quantified for each sample relative to a normalization transcript using the PerfeCTa SYBR Green FastMix (Quantabio) in 96-well PCR plates (Hard-Shell 480 PCR Plates, Bio-Rad) measured in a LightCycler 480 (Roche). Individual 10 µl reactions consisted of 5 µl FastMix, 1 µl target-specific primer pairs (0.5 µl 10 µM forward primer and 0.5 µl 10 µM reverse primer) (Table 4), 3 µl DEPC-treated water and 1 µl cDNA, mock cDNA or water. Plates were sealed with optically clear film (PlateSeal). A standard SYBR green PCR programme was used with modifications: 95 °C for 5 min, 45 cycles of 95 °C for 10 s, 55 °C for 10 s and 72 °C for 20 s with a single fluorescence acquisition. A melting curve was conducted directly following the PCR programme to confirm the presence of individual species amplified in each well. The melting curve programme was 95 °C for 5 s, 65 °C for 1 min and ramp up to 97 °C by 0.11 °C s^−1^ with continuous fluorescence acquisition. *ALG9* was used as a normalization transcript. All samples were measured with three technical replicates. Mock cDNA (prepared without the addition of reverse transcriptase) and water were used as negative controls.

**Table 4.** qRT-PCR primers

Gene_pair#*	Size	Efficiency	Forward primer sequence 5′–3′	Reverse primer sequence 5′–3′
ACS1_1	188	nt†	TAGTTGGTCCACCGCAACAG	TGGTCACTTTCTCCTGCTAG
ACS2_1	194	nt	CAACGCTACTGAAGGTGATG	TGTTCGGCTTCGTTAGAAGC
ADH1_1	139	2.03	CAAGGCTATGGGTTACAGAG	GTCAGTGGCCTTTAGAACAG
ADH2_1	129	2.02	CGAACGGTACTGTTGTCTTG	GCTTCTCTGGTATCAGCTCTG
ADH3_1	150	nt	CCCTCATGGTGTCATTAACG	GATGGACTTCACCACATGAG
ADH4_1	130	nt	TGGCAGGTATGGCTTTCAAC	GTTGGCCTCTTGAACATGAG
ADH5_1	120	nt	TAATGGCGGTTCTCATGGAG	GTAAGCATGAGCTGGCATAC
ALD2/3_1	205	nt	TTGGTGGAGCTAAAGGCTAC	CATGTGCGCTTTCTTGACATC
ALD4_1	183	1.98	CCCTGTTGTCACTGTAACC	AACTGCGTGGTGGAAATCG
ALD5_1	123	nt	CGATATTAACAAGGCTGTTGATG	ACGGCCAATACCTGACTGG
ALD6_1	140	2.01	GCTAACTTCCAAGGTGCTATC	GGTTGGTCTGATGAAGTAACC
ALG9_1	123	1.98	CTGAATTTCCCACTGCCTGTG	CCTCTTTGTGAGGTTGTTGAG
ALG9_2	142	2.02	GGATAGTGGCTTTGGTGAAC	CAGGAAAGAACTTGGGAAGTG
ARO10_1	168	2.05	CACTCAAACGCTCACATCAATG	TAGTGTAGCCGTTATTGTTCC
ATF2_1	245	1.73	ATTCGACATTCCCGAAGGTG	TAGTACGGCTTGGTCGTATC
BAT2_1	116	1.87	AGACTCGAACCAAGTGAATGG	AATCGCAGCAGTACCAGAAC
FPS1_1	172	nt	CATCGGACCTGAGTGACTTC	ACCACCAAACGTTCTTTTGC
GPD1_1	152	1.94	CTTGCCTGGCATCACTCTAC	CTGACGTGTGAATCAACATG
GUT1_1	116	nt	GCAAATTCAAGCCGATATCTTAG	TCGTTCACATCCTTGAAAGC
GUT1_2	119	2.21	ATATCACCAGAGGCTCATCC	CTTCAGCCAACCTTTGGATC
IRC7_1	145	nt	GGCTCGGAAATCGAGATGAG	CAGACCAGCTCTTGTGAACC
LEU4_1	138	nt	CAATGACGAACAGTACCAAGC	GATTGGACCATTACCTGTACC
MAL12/32_1	147	nt	CCTACATCGAGAATCACGATC	CCTATCTCCTGACCTTGATAG
MED15_1	297	2.02	AGATGCTTACGTCATGCACTATCC	GGGTTGCCGACATCCATATTAG
MET10_1	109	nt	ACTCAATTGCCTCCTCTCAG	CTTAGAAGCTTGACCGTACC
PDA1_1	215	nt	ATCCAAGAACGATCCAATTGC	AATTTGGCTTCTGGAGGAGG
PDB1_1	148	nt	TATCCCCTGAGTTCACCTTG	GATAACTTCTGCAGAGACACC
PDC1_1	227	2.01	TCTCCACCATGATCAGATGG	TGTCGTTGAAAGACTTGTCTTG
PDC6_1	217	2.03	TCTCCACCATGATCAGATGG	CTCTGAATCAGTGGTTAAGGC
PTR2_1	135	1.94	CTTTTTCAGAGATCTTTGCCTCTATC	GACAATGCACAACCGATTGC
PTR2_2	122	nt	GTTTGTTCTGGTTGTGCTTCAG	CAATATCGTTAGCTTTAGGTGCG

* Pair number for multiple primer pair sets designed for a given gene.

† nt, not tested.

The relative abundance of target transcripts was determined by calculating the crossing point (CP) value for each reaction, which reflects the number of cycles of amplification required for the SYBR green signal to exceed a threshold value set to eliminate background noise. CP values were calculated using the Second Derivative Maximum method implemented in the LightCycler 480 Software (Roche). The average CP for technical replicates amplifying the target transcript with a specific RNA sample was normalized to the average CP for technical replicates amplifying the normalization transcript. This ratio was then compared across samples as a depiction of relative abundance of the target transcript in each sample.

### RNA sequencing

Individual fermentation reactions in 5 ml supplemented WGJ were prepared as previously described for two biological replicates each of the *URA3*-marked *med15Δ* strain and integrants of the LAB, WY7, WY15 and WY23 *MED15* alleles. Weight loss was monitored, and yeast were pelleted at the 35% weight loss point (~37 h for *MED15* strains and 48 h for *med15Δ* strains). RNA was extracted as described above. RNA sample quality was assessed using an Experion automated electrophoresis system with a HighSens chip using a standard protocol (Bio-Rad). cDNA libraries were prepared using the Illumina Stranded mRNA Prep protocol (Illumina). A total of 150–200 ng of total RNA per sample and 13 cycles of amplification were used. The Illumina Dual Index Adapters set A (Integrated DNA Technologies) was ligated to the cDNA.

cDNA library quality control and sequencing were performed by the Iowa Institute for Human Genetics (IIHG) Genomics Division. cDNA libraries were quantified using fluorometry with PicoGreen. The integrity of the cDNA libraries was determined using an Agilent BioAnalyzer DNA chip. A KAPA qPCR was used to get precise quantification prior to loading samples for sequencing. The pooled libraries were run with the 2×150 bp protocol on one lane of an S Prime flow cell in an Illumina NovaSeq.

### RNA-Seq data analysis

Sequencing data was processed using a manually curated pipeline of programmes implemented in the IIHG instance of the Galaxy platform, a web-based computational workbench [41]. Adapter sequences were trimmed from reads using TrimGalore! (Galaxy tool version 0.6.3). The adapter sequence was auto-detected. Reads shorter than 50 bp after trimming were discarded. Any reads orphaned at this point were also discarded to maintain sets of fully paired reads. Paired-end reads were then mapped to the S288C reference genome (sacCer3) using Bowtie2 (Galaxy tool version 2.3.4.2) [42]. Default parameters for paired-end reads were used, the maximum paired-end length was set to 5,000 and dovetailing allowed to maximize the number of mapped reads. Counts were quantified using featureCounts (Galaxy tool version 1.6.3) [43] to tally the number of reads mapping to annotated ‘genes’ in the S288C reference general feature format file (version R64-3-1). Count normalization and differential expression analysis was done using DESeq2 (Galaxy tool version 2.11.40.2) [44] using default parameters with multiple testing correction using Benjamini–Hochberg method with a false discovery rate of 5%. Biological replicates were compared at the level of normalized counts with correlation analysis (Fig. S1, available in the online Supplementary Material).

Alternative mapping and differential expression analyses included the use of limma (version 3.60.6), kallisto (version 0.46.1) and sleuth (version 0.30.2) [45–47]. Limma was used as a potentially less conservative alternative to DESeq. The featureCounts table from the Bowtie2 method was transformed using voom [48] prior to linear model fitting with limma using default methods. kallisto was used as an alternative method to increase the percentage of uniquely alignable reads. A kallisto index was prepared with default settings using the SacCer3 reference transcriptome. Trimmed reads were pseudoaligned using kallisto quant with default settings and 100 bootstraps. Differential expression analysis was done on uniquely aligned and pseudoaligned reads. kallisto abundance.h5 outputs were prepared for DESeq2 using tximport [49]. The transcript-to-gene associations were determined using biomaRt [50] ‘refseq_dna’ and ‘ensembl_gene_id’ attributes. Alternatively, kallisto abundance.h5 output files were used directly as inputs for sleuth differential expression analysis. A sleuth object was created using sleuth_prep with the extra_bootstrap_summary argument set to TRUE to take advantage of the bootstrapping done while running kallisto. The effect of the strain genotype on expression patterns was modelled using sleuth_lrt. Differential expression for each strain genotype relative to the lab strain was determined using sleuth_wt.

Fig. S2 (A–D) compares the DRGs identified with the four methods we tested. Venn diagrams showing overlap of genes with adjusted *P*-values<0.05 for each of four methods [Bowtie2 ->DESeq2 (BowDE), Bowtie2 ->limma (BowLi), kallisto ->DESeq2 (KalDE) and kallisto ->sleuth (KalSl)] for each of the four comparisons described as part of this work (*MED15*_LAB_ vs *med15*Δ, *MED15*_WY7_ vs *MED15*_LAB_, *MED15*_WY15_ vs *MED15*_LAB_ and *MED15*_WY23_ vs *MED15*_LAB_) emphasize that the Bowtie2-DESeq2 pipeline performs as well as any of the other pipelines in terms of identifying DRGs. Pearson correlation matrices of the differential expression statistics (DESeq2 stat values, limma t-values and sleuth b-values) for the shared set of genes for each of four methods for the four comparisons are shown in Fig. S2 (E–H).

### Data representation

Volcano plots of differentially expressed genes were prepared using VolcaNoseR [51]. Venn diagrams of overlapping differentially expressed genes were prepared using VennDiagram [52]. Gene ontology (GO)-enrichment analyses were done using AllianceMine [53]. Metabolic pathway enrichment analyses were done using BioCyc [54].

### GO enrichment

Differentially expressed genes were input as new lists on AllianceMine [53]. Biological process GO enrichment was done automatically. Enriched GO terms with Bonferroni adjusted *P*-values<0.05 were obtained. For each term, a ‘Med15 Regulated’ frequency was calculated by dividing the number of DRGs with that GO term over the total number of DRGs in the list. An ‘All Genes’ frequency was determined by dividing the number of yeast genes with that GO term by the total number of yeast genes in the ALL_Yeast_Genes list (*n*=7,173). Redundant or highly related enriched GO terms were manually removed.

### Gene set enrichment analysis

Pathways enriched in differentially expressed genes, in comparisons of *MED15*_WY_ alleles with *MED15*_LAB_*,* were identified using fgsea [55]. Wald statistics for differential expression from the DESeq2 analyses were used to determine enrichment among 1,234 yeast pathways compiled in the ConsensusPathDB [56]. These included pathways from BioCyc, KEGG and Reactome among others. For each comparison, pathways were excluded from the analysis if there were five or fewer genes with Wald statistics within that pathway. Pathways with adjusted *P*-values less than 0.01 were considered significantly enriched. Venn diagrams of overlapping enriched pathways were created using VennDiagram [52]. Heatmaps for gene sets that correspond to specific pathways were created using pheatmap [57] for log-transformed normalized counts.

### Primers

All primers were synthesized by Integrated DNA Technologies and are listed in Tables 3 and 4. Primer pair efficiency was measured by qRT-PCR analysis of serial dilution of control RNA.

## Results

### Fermentation gene expression is affected in the *med15Δ* strain

**Fig. 1.**
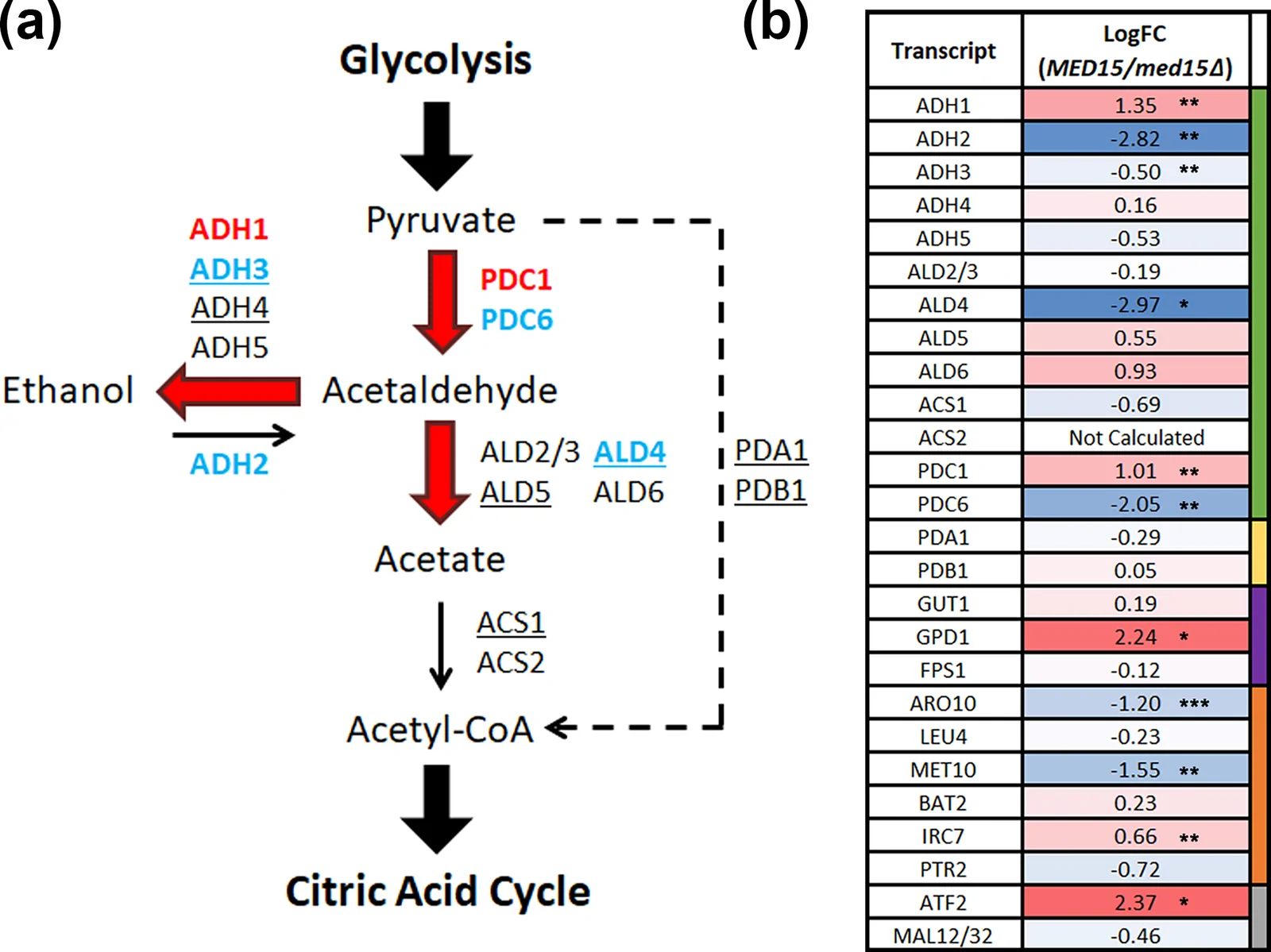
Influence of Med15 on expression of alcoholic fermentation pathway genes. (**a**) Summary of predicted impact of *med15Δ* on ethanol metabolism. Tested metabolic genes labelled according to changes in expression in *med15Δ* relative to expression in *MED15* yeast. Gene names in red are upregulated (activated) by *MED15*, gene names in blue are downregulated (repressed) by *MED15*, and gene names in black are not significantly regulated by *MED15*. Underlined genes are mitochondrial. (**b**) Gene expression log2 fold-change measured by qRT-PCR normalized to *ALG9* in *MED15* relative to *med15Δ* grown in YPD with three to six technical replicates. One outlier was removed with a Grubb’s test. Relative expression values for genes with shades of red representing extent of activation by *MED15*, and shades of blue representing extent of repression by *MED15*. Significance was determined using t-tests with a Bonferroni correction for multiple testing (* <0.05; ** <0.01; *** <0.001). Genes are grouped by function: alcoholic fermentation (green), respiration (yellow), glycerol metabolism (purple), amino acid metabolism (orange) or other (grey).

We previously observed that a *med15Δ* lab strain is defective in the fermentation of grape juice [1]. We hypothesized that this fermentation defect is the consequence of changes in the expression of metabolic genes involved in the production or consumption of ethanol. To determine the role of Med15 in the regulation of relevant fermentation genes, we first assembled a list of target genes by reviewing existing *med15Δ* microarray datasets [36, 37, 58] and identifying genes with previously described roles in ethanol metabolism and wine or beer fermentation [59–61]. We then used qRT-PCR to evaluate their expression in standard growth media. Genes we found to be regulated by *MED15* had roles in ethanol metabolism and fermentation, including three (*PDC1*, *ACS1* and *ADH2*) that had not previously been recognized as *MED15* targets. Both positive regulation by Med15 (lower expression in the deletion strain) and negative regulation by Med15 (higher expression in the deletion strain) was observed. For example, the ADHs *ADH1* and *ADH3,* which facilitate the conversion of acetaldehyde to ethanol, were reduced in the *med15*Δ strain, whereas *ADH2*, which catalyses the reverse reaction (ethanol to acetaldehyde), was significantly upregulated (Fig. 1). Similarly, *ALD4*, which is involved in the conversion of acetaldehyde to acetate, is increased in the *med15*Δ strain (Fig. 1) indicating that Med15 typically represses the mitochondrial, glucose-repressed *ALD4*. *PDC1*, the major isoform responsible for converting pyruvate to acetaldehyde during fermentation, was downregulated in the *med15*Δ strain, whereas *PDC6*, a minor isoform typically expressed under stress conditions, was upregulated indicating that Med15 promotes *PDC1* and represses *PDC6* expression to reinforce a fermentative metabolic profile. Together, the observed transcriptional effects are consistent with Med15 playing a central role in fermentation by regulating key enzymes that drive ethanol production and stress-related metabolic shifts.

### The *MED15* transcriptome is highly specialized during alcoholic fermentation

**Fig. 2.**
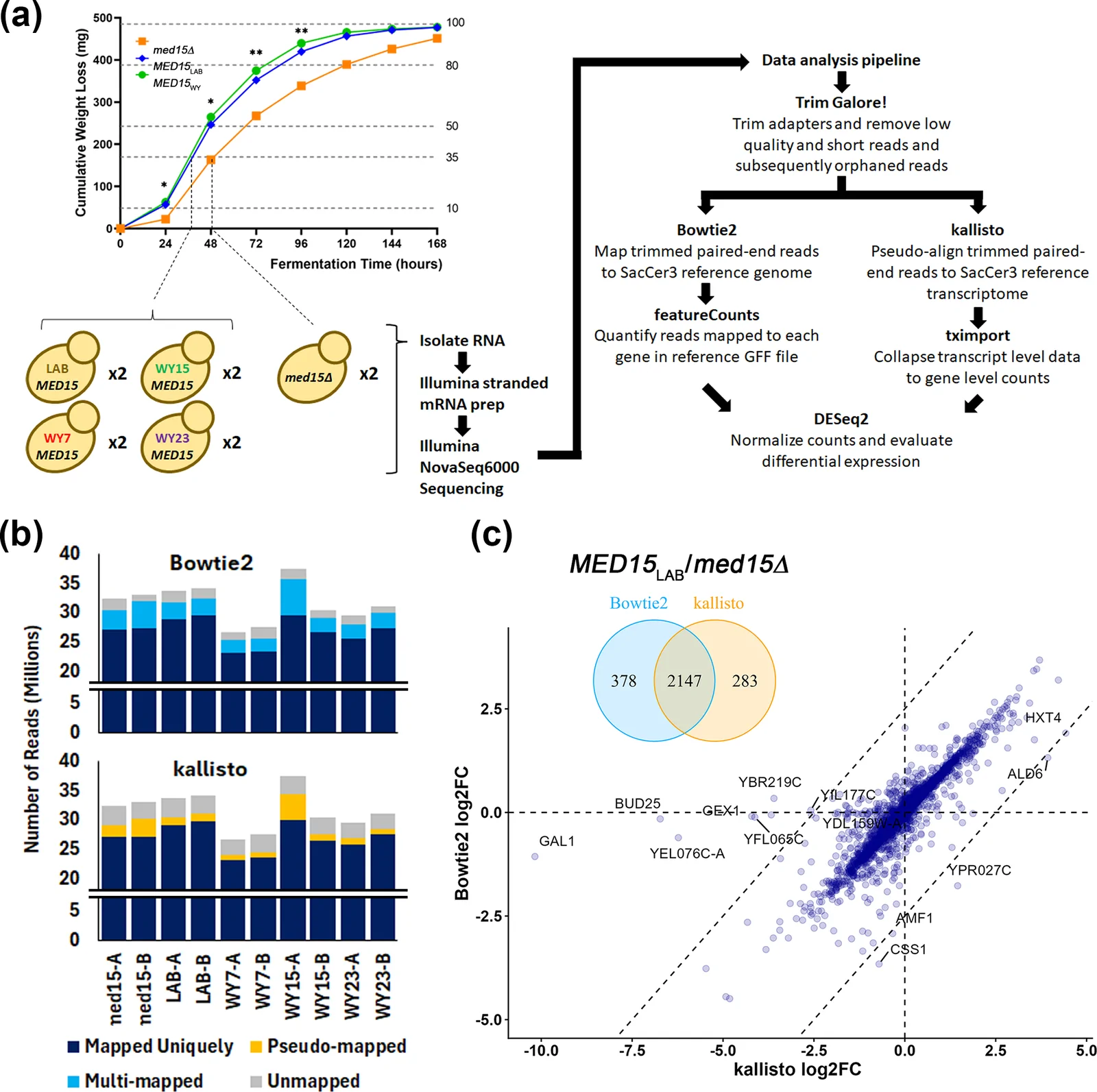
WGJ RNA sequencing experimental setup and summary results. (**a**) RNA sequencing experimental overview with a description of two alternative analysis pipelines. (**b**) Summary mapping results from Bowtie2 [42] and kallisto [45] for each sample. Counts were then used as input for DESeq2 [44] to determine differential expression. (**c**) Venn diagram of DESeq2 determined DRGs (adjusted *P*-value<0.05) for Bowtie2 and/or kallisto methods. Scatter plot showing the relationship between DESeq2 log2 fold-change values for Bowtie2 and kallisto methods. 90% of genes (5117/5678) have log2 fold-change values that differ by less than 0.255 between the two methods. Genes with extreme variation (greater than 2.5 difference in log2 fold-change) are labelled.

To determine the global impact of *med15Δ* on gene expression during alcoholic fermentation rather than in standard growth media (YPD or SC at 2% glucose) as in the datasets discussed above, an RNA sequencing experiment was conducted for cells grown in WGJ (20% glucose) at the 35% wt loss fermentation benchmark (Fig. 2a) [1, 8]. Note that weight loss benchmarks rather than time were used to coordinate sample collection in this experiment because the *med15Δ* strain grows and ferments much slower than strains with an intact *MED15* gene. This global analysis was expected to identify all affected genes including any that we had not anticipated, and the fermentation conditions were expected to have a substantial impact on gene expression.

Due to the relatively high number of multi-mapped reads in our dataset using Bowtie2 [42], we also employed kallisto [45], an RNA-Seq quantification programme that incorporates pseudoalignment of multi-mapped reads (Fig. 2b) and thus increased the number of uniquely mapped reads to ~90%. The increase in mapped reads did not substantively change the DRG set. Seventy-nine percent of genes identified as differentially regulated were common regardless of the read-mapping strategy, and the extent of gene regulation, as expressed by the log2 fold-change, was also similar (Fig. 2c). Genes whose counts increased by an average greater than 10,000 reads using the kallisto method included members of large gene families like the small and large ribosomal proteins as well as translation factors (~50%), glycolysis genes (~15%), transport/nutrient uptake (~10%), subtelomeric genes (~10%) and chromatin/stress genes (~5–10%) (Table S1). These were not among the very few genes whose extent of regulation was drastically affected (reside outside the diagonals) in the kallisto dataset (Fig. 2c), but instead these genes had proportional increases in mapped reads across samples using kallisto.

**Fig. 3.**
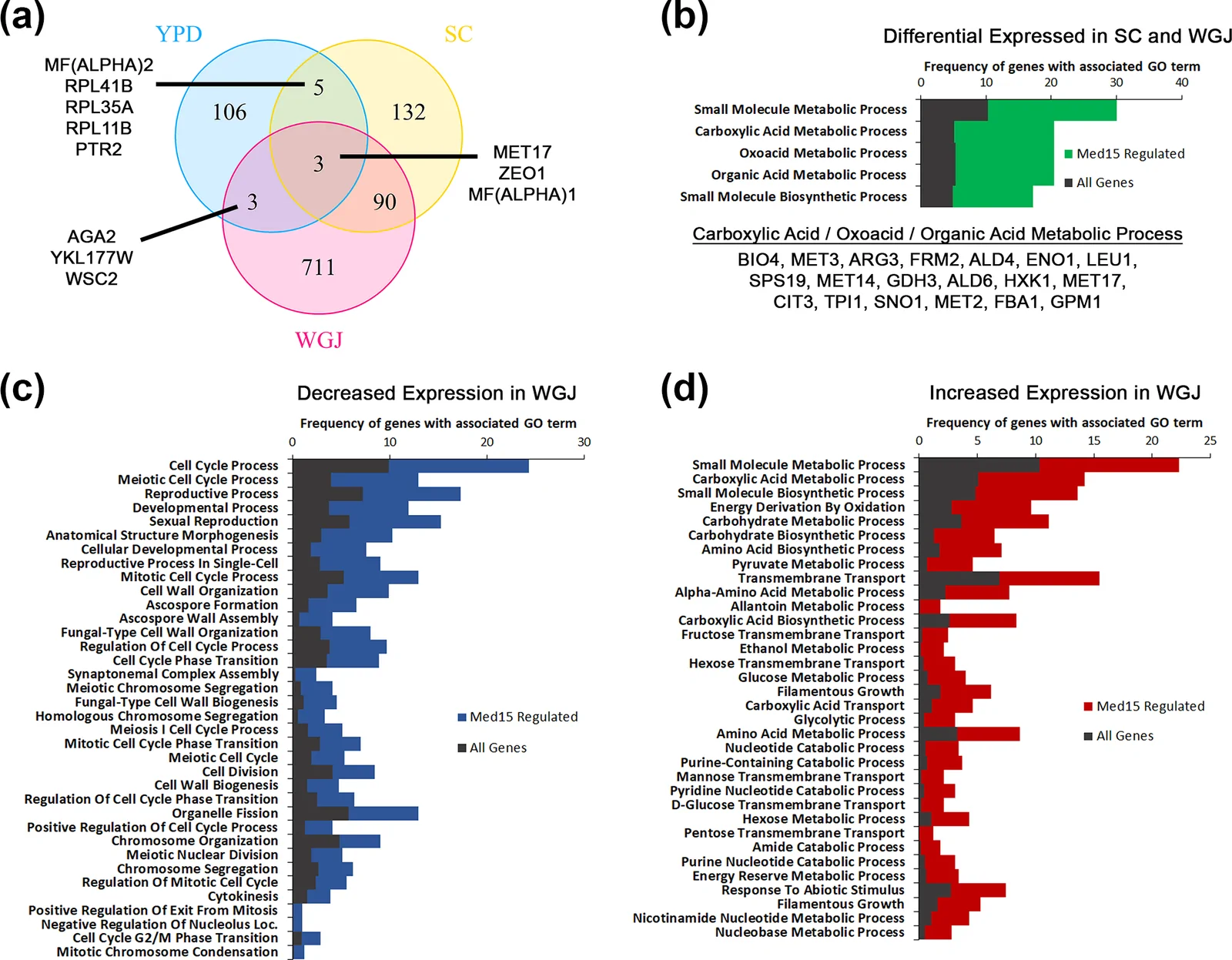
Condition-specific and general metabolic pathways regulated by *MED15*. (**a**) Overlap between significant (log2 fold-change>1 or <−1 and adjusted *P*-value<0.05) differentially regulated genes (DRGs) in comparisons of the *MED15* and *med15Δ* strain for log-phase growth in YPD [36] as reanalysed by Reimand *et al*. [38], log-phase growth in SC [37], and WGJ fermentation (this study) using the combined Bowtie2-DESeq2 and kallisto-DESeq2 pipeline gene lists. (**b**) Gene ontology (GO) enrichment using AllianceMine [53] of the 93 gene overlap between the WGJ and SC groups from (**a**). All enriched GO terms shown have a Bonferroni adjusted *P*-value<0.05. The DRGs shared by the closely related carboxylic acid metabolic process (most specific) GO terms are displayed. (**c**) GO enrichment (as for b) of the 485 genes with decreased expression in the *MED15*_LAB_ strain relative to deletion for the Bowtie2-DESeq2 and/or kallisto-DESeq2 methods. All enriched GO terms shown have a Bonferroni adjusted *P*-value<0.05. Closely related GO-terms were manually removed. (**d**) GO enrichment (as for b) of the 323 genes with increased expression in the *MED15*_LAB_ strain relative to deletion for the Bowtie2-DESeq2 and/or kallisto-DESeq2 methods. All enriched GO terms shown have a Bonferroni adjusted *P*-value<0.05. Closely related GO-terms were manually removed.

In this analysis, we found 807 genes that were differentially regulated in a comparison of WT and the *med15Δ* strain under the WGJ condition (log2 fold-change >1 or <−1 and adjusted *P*-value<0.05, Bowtie2-DESeq2 and/or kallisto-DESeq2 DRGs). 0.7% are in common with DRGs identified in the YPD microarray dataset [36, 38], and 11.5% of the WGJ DRGs are in common with DRGs identified in the SC microarray dataset [37] (Fig. 3a). The 93 DRGs in common between the WGJ and SC lists were enriched (adjusted *P*-value<0.05) for GO terms related to core biosynthetic metabolism, including genes involved in sulphur and nitrogen assimilation, glycolytic flux and redox balancing under fermentative conditions (Fig. 3b).

Among the 485 genes negatively regulated by Med15 (significantly lower expression in *MED15* vs *med15Δ*) within the WGJ environment the enriched GO terms included: sporulation, cell cycle, DNA replication, cell division, checkpoints, exit from mitosis and cell wall organization (Fig. 3c). A total of 323 genes that were positively regulated by Med15 in WGJ were enriched in amino acid and nucleotide (nt) biosynthesis and transport and response to stress (Fig. 3d) as well as carbohydrate transport (pentose, hexose, glucose, fructose and mannose) including several glucose transporters [high-affinity (*HXT4*, *HXT6* and *HXT7*) and low-affinity (*HXT1* and *HXT3*)]. Interestingly, two additional *HXT* genes, *HXT10* and *HXT14,* were present in the negatively regulated gene list.

**Fig. 4.**
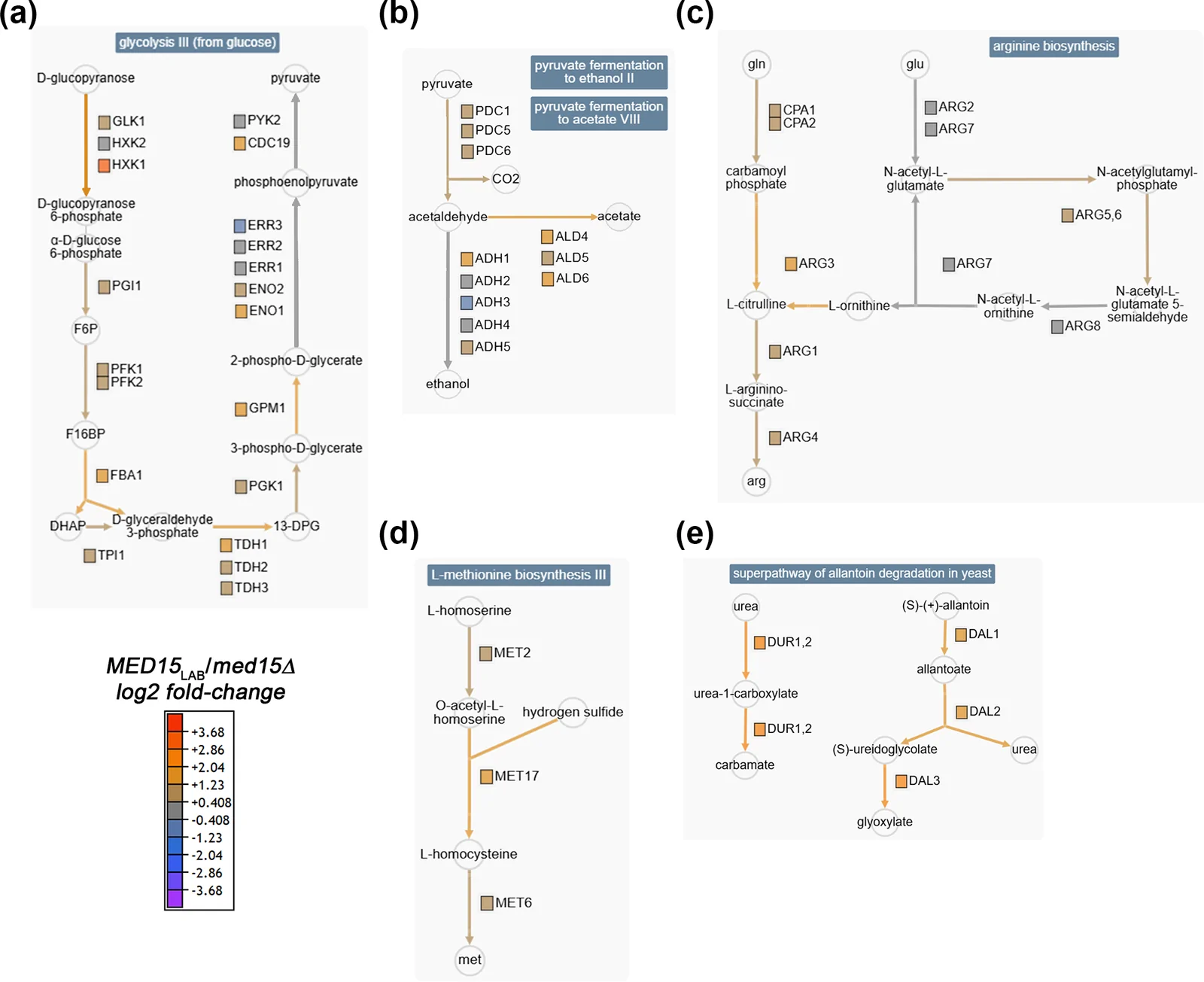
Metabolic pathways regulated by Med15 in WGJ. (**a**) Glycolysis; (**b**) pyruvate fermentation; (**c**) arginine biosynthesis; (**d**) methionine metabolism and (**e**) allantoin metabolism. Pathways are generated using BioCyc [54]. The colour of the edges (arrows) are approximations of the sum of the effects on the individual steps (enzymes) in the pathway using Bowtie2-DESeq2 differential expression data. See key for the relative Med15 dependence of each enzyme in the pathway.

To achieve more nuanced insight into the *MED15*-dependent metabolic profile during WGJ fermentation, we analysed the distribution of *MED15*-regulated genes across all yeast metabolic pathways using the BioCyc database [54] with the entire WT vs *med15Δ* WGJ fermentation dataset as input (Bowtie2-DESeq2 – including positively regulated, negatively regulated and genes not regulated by *MED15*). In addition to glycolytic and ethanol metabolic pathways (Fig. 4a, b), the arginine biosynthetic pathway was specifically affected during WGJ fermentation (Fig. 4c). A similar enrichment was seen for genes involved in the methionine biosynthesis (Fig. 4d). Allantoin catabolism was another significant pathway regulated by *MED15* during fermentation, with all allantoin catabolism enzymes (*DAL1*, *DAL2*, *DAL3* and *DUR1,2*) affected (Fig. 4e).

**Fig. 5.**
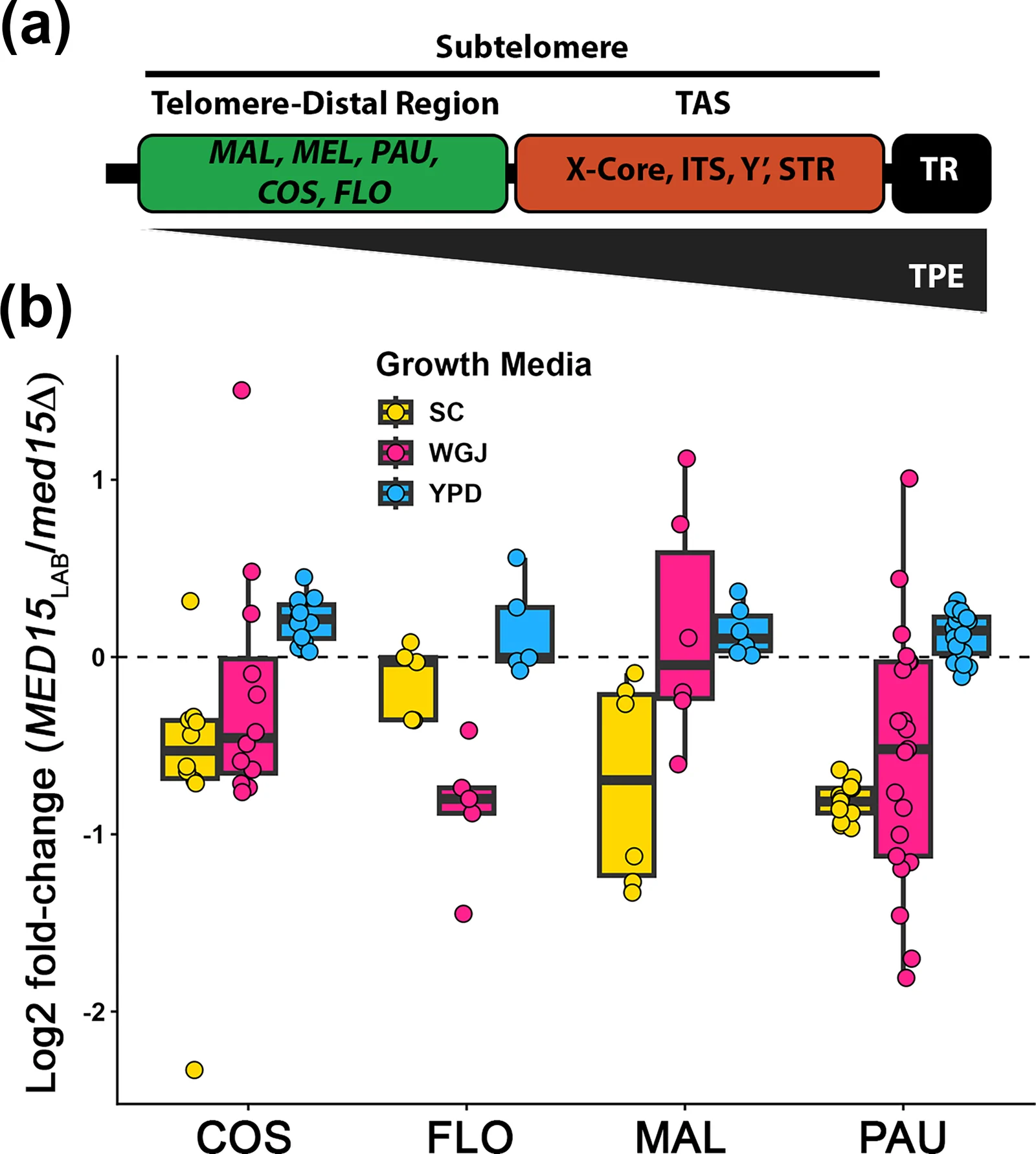
*MED15* regulates silencing of subtelomeric genes during WGJ fermentation. (**a**) Cartoon depiction of *S. cerevisiae* telomeres and subtelomeric features. (**b**) Differential expression of subtelomeric genes of each indicated class (COS, FLO, MAL, PAU) during WGJ fermentation, or during growth in SC or YPD media. Log2 fold-change values for *MED15*_LAB_/*med15Δ* are plotted for WGJ (Bowtie2-DESeq2), SC and YPD [36, 37, 58]. Data were as previously described without further processing or filtering by *P*-value. All representative genes for the four gene families were selected from the three data tables. ITS, interspersed telomeric repeats; STR, subtelomeric repeat sequences; TAS, telomere-associated sequence; TPE, telomere position effect; TR, telomeric repeats.

Because published reports show that *MED15* affects silencing at telomeres during normal growth [62, 63], subtelomerically localized genes were evaluated in the WGJ fermentation dataset. We evaluated the distal region of yeast telomeres, which contains genes belonging to a small number of gene families, including *MAL*, *MEL*, *PAU*, *COS* and *FLO* genes (Fig. 5a), although the S288C lab strain used here lacks *MEL* genes [64] and only has two partially functional *MAL* gene regulons [65, 66]. Most *PAU* genes (12/21) and *COS* genes (8/12) and all *FLO* genes (5/5) were significantly differentially regulated by *MED15* under WGJ fermentation conditions (adjusted *P*-value<0.05, Bowtie2-DESeq2 DRGs). Virtually all differentially regulated *FLO*, *PAU* and *COS* genes (except for *PAU9* and *COS111*) were more highly expressed in the *med15Δ* strain under WGJ fermentation conditions, a pattern also seen in the SC transcriptome. This pattern suggests that during WGJ fermentation (and growth in SC media) *MED15* promotes or helps maintain silencing of subtelomeric gene families. This is in contrast to the expression pattern of subtelomeric genes during log-phase growth in YPD in which the average log2 fold-change across gene families is modestly elevated in the WT strain [36, 37, 58].

### *MED15*_WY_ - *MED15*_LAB_ comparisons reveal subtle effects on the fermentation transcriptome

**Fig. 6.**
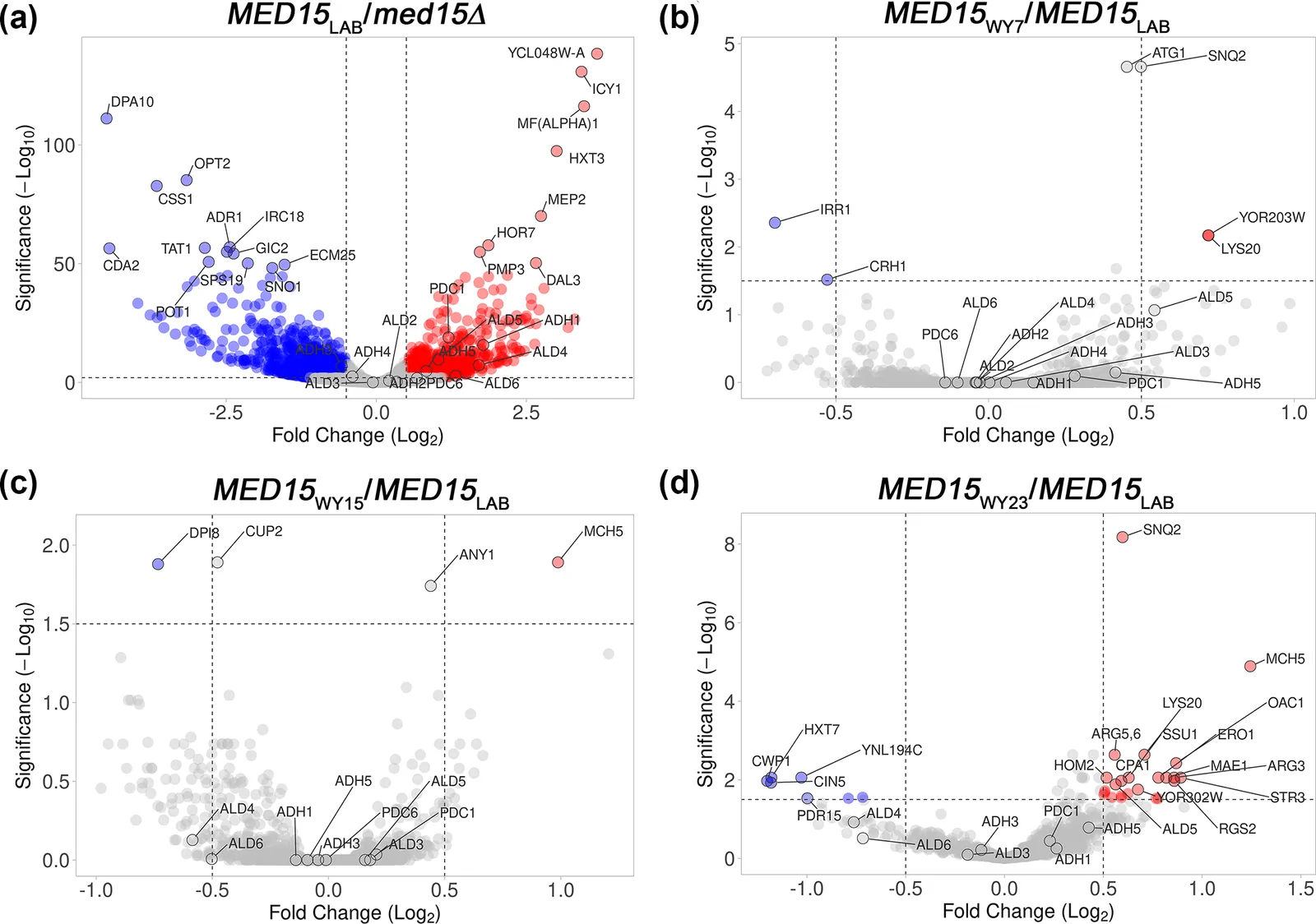
Fermentation metabolic gene expression in *MED15*_WY23_ vs *MED15*_LAB_. (**a–d**) Volcano plot of DRGs (Bowtie2-DESeq2) between strains expressing *MED15_LAB_* and deletion (Δ) (**a**), *MED15*_WY7_ (**b**), *MED15*_WY15_ (**c**) and *MED15*_WY23_ (**d**) are shown. Highlighted in each plot are genes that exceed both log2 fold-change and *P*-value thresholds represented by vertical and horizontal lines. Genes that are indicated in blue are negatively affected by the WY allele, and genes in red are positively affected by the WY allele. Each coloured gene in the *MED15*_WY7_, *MED15*_WY15_ and *MED15*_WY23_ plots is annotated. The most significantly regulated genes in the *MED15*_LAB_/*med15*Δ plot are annotated. Additional annotations are for fermentation genes shown to be regulated (in YDP) as in Fig. 1.

To investigate the impact of WY *MED15* alleles during WGJ fermentation, we examined the impact of three different WY *MED15* alleles integrated into the lab strain genome as the sole *MED15* gene. RNA-Seq analysis was conducted at 35% weight loss (Fig. 2). Overall, the fermentation genes (labelled in Fig. 6) were not significantly affected (Bowtie2-DESeq2 data). Only the *ALD* genes displayed absolute log2 fold-change values greater than 0.5 (Fig. 6b-d) and among these, only *ALD5* was significant and this was true only in *MED15*_WY23_ (Fig. 6d). Thus, the expression of most fermentation genes was unchanged in WGJ environment regardless of *MED15* allele suggesting that the enhanced fermentation phenotypes in strains with *MED15*_WY_ alleles [1] is rooted in expression differences in other classes of genes.

**Fig. 7.**
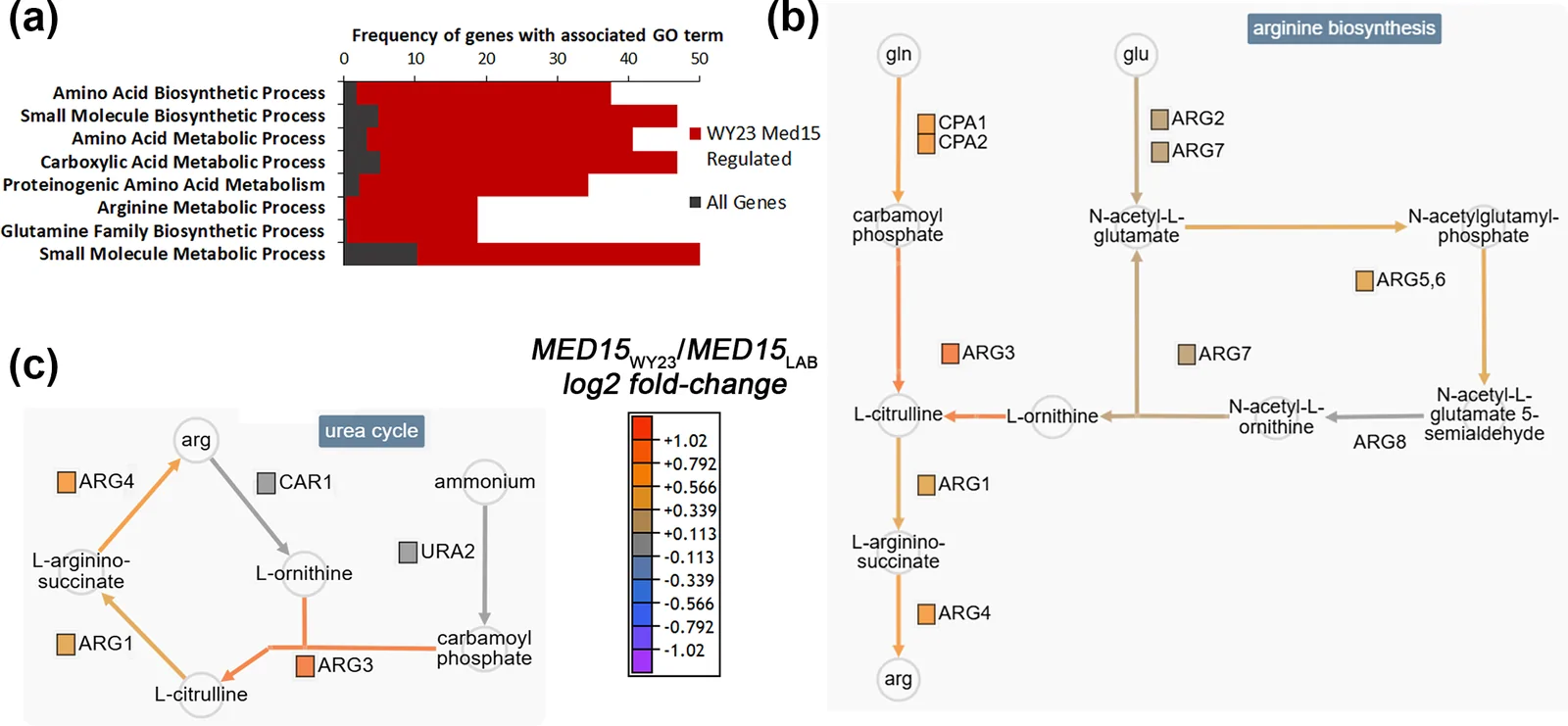
*MED15*_WY23_ differentially modulates the expression of metabolic genes during WGJ fermentation. (**a**) GO enrichment using AllianceMine [53] as in Fig. 3 of the 32 genes with increased expression in the *MED15*_WY23_ relative to the *MED15*_LAB_ strain in the WGJ fermentation environment for the Bowtie2-DESeq2 and/or the kallisto-DESeq2 methods. All enriched GO terms shown have a Bonferroni adjusted *P*-value<0.05. Closely related GO terms were manually removed. (**b, c**) Metabolic pathways for arginine biosynthesis (**b**) and urea cycle (**c**). Metabolic pathway analysis conducted with BioCyc [54]. The colour of the edges are approximations of the sum of the effects on the individual enzymes in the pathway using Bowtie2-DESeq2 differential expression data.

To investigate the transcriptomic basis for improved WGJ fermentation in strains differing only in their *MED1*5 allele [1], we applied GO and Pathway enrichment analyses for the DRGs from comparisons of the *MED15_WY_* and *MED15_LAB_* transcriptomes. A comparison of *MED15*_WY23_ and *MED15*_LAB_ strains in the WGJ environment revealed 42 DRGs (log2 fold-change>0.5 (*n*=32) or <−0.5 (*n*=10) and adjusted *P*-value<0.05, Bowtie2-DESeq2 and/or kallisto-DESeq2 DRGs) (Fig. 7 and Table S2). GO enrichment analysis of genes with increased expression with *MED15*_WY23_ (*n*=32) pointed to an enrichment in amino acid metabolism with a specific effect on the arginine biosynthetic pathway. We also checked the genes of the urea cycle, which intersects with the arginine pathway at ornithine, a metabolic pivot for bidirectional nitrogen management. The effect of *MED15*_WY23_ on both arginine and urea cycle genes suggests an effect on nitrogen metabolism (Fig. 7). *MAE1* (malate dehydrogenase) expression was also significantly upregulated in the *MED15*_WY23_ strain, alongside that of other enzymes in the gluconeogenesis pathway (Fig. 6d and Table S2).

**Fig. 8.**
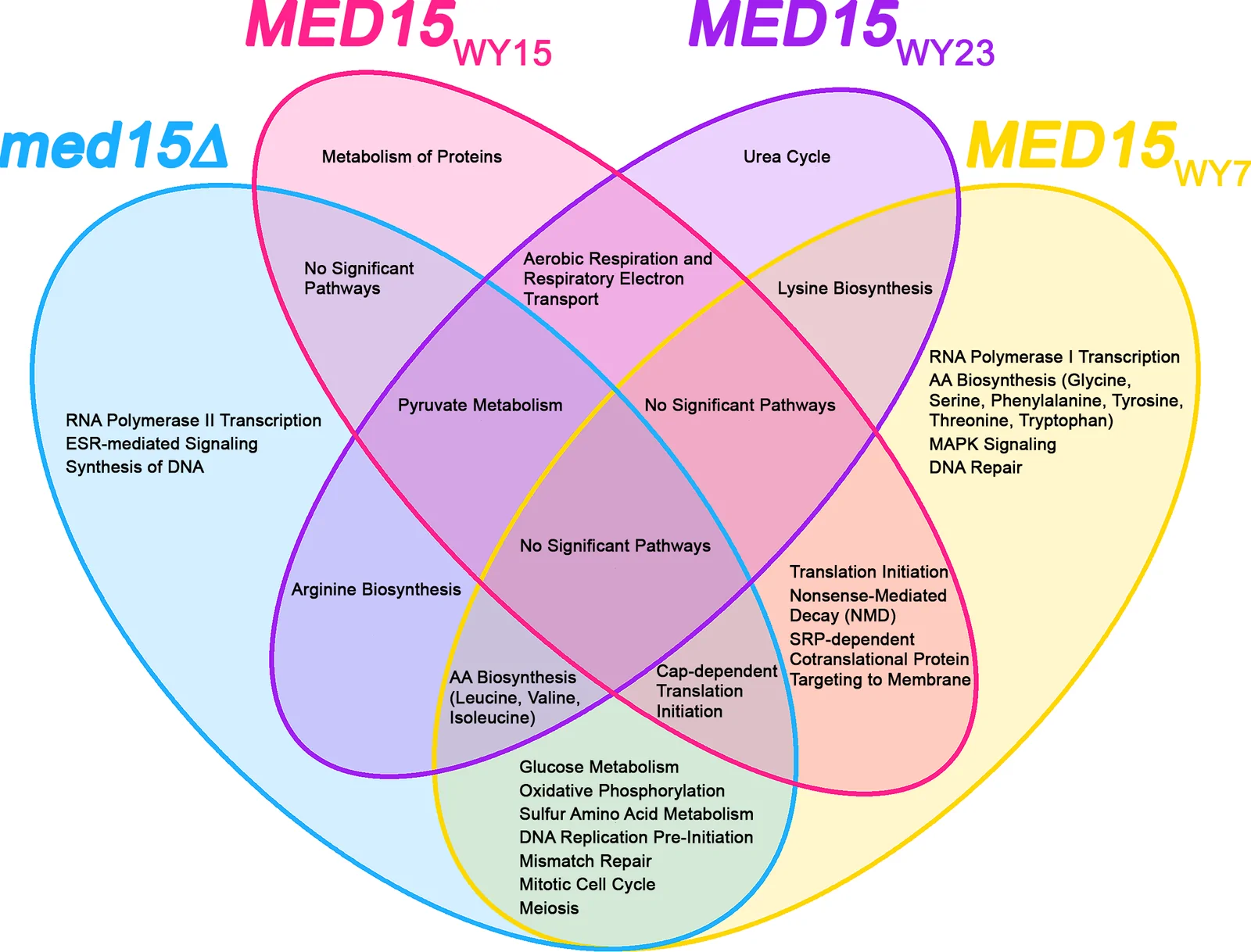
Venn diagram depicting shared and unique pathways that are enriched in S288C-based yeast strains with different *MED15*_WY_ alleles. Genes regulated by *MED15* presence/absence in the lab strain are categorized in the blue segment of the diagram. Some of the same gene categories are affected in comparisons of strains with *MED15*_WY_ alleles compared to the *MED15*_LAB_ allele. For example, arginine biosynthesis genes are affected by the absence of *MED15* and by the *MED15*_WY23_ allele. Other affected gene categories are uniquely detected in comparisons of *MED15*_LAB_ strains to multiple *MED15*_WY_ alleles. For example, lysine biosynthesis is detected in *MED15*_WY23_ and *MED15*_WY7_ comparisons to *MED15*_LAB_ but is not among genes detected in a comparison between *MED15*_LAB_ and deletion strains. Finally, some gene categories (e.g. urea cycle genes) are unique to the *MED15*_WY23_ allele. This points to the existence of different gene expression patterns that may account for the enhanced fermentation characteristics exhibited by strains with *MED15*_WY_ alleles and to the WY strains themselves.

**Fig. 9.**
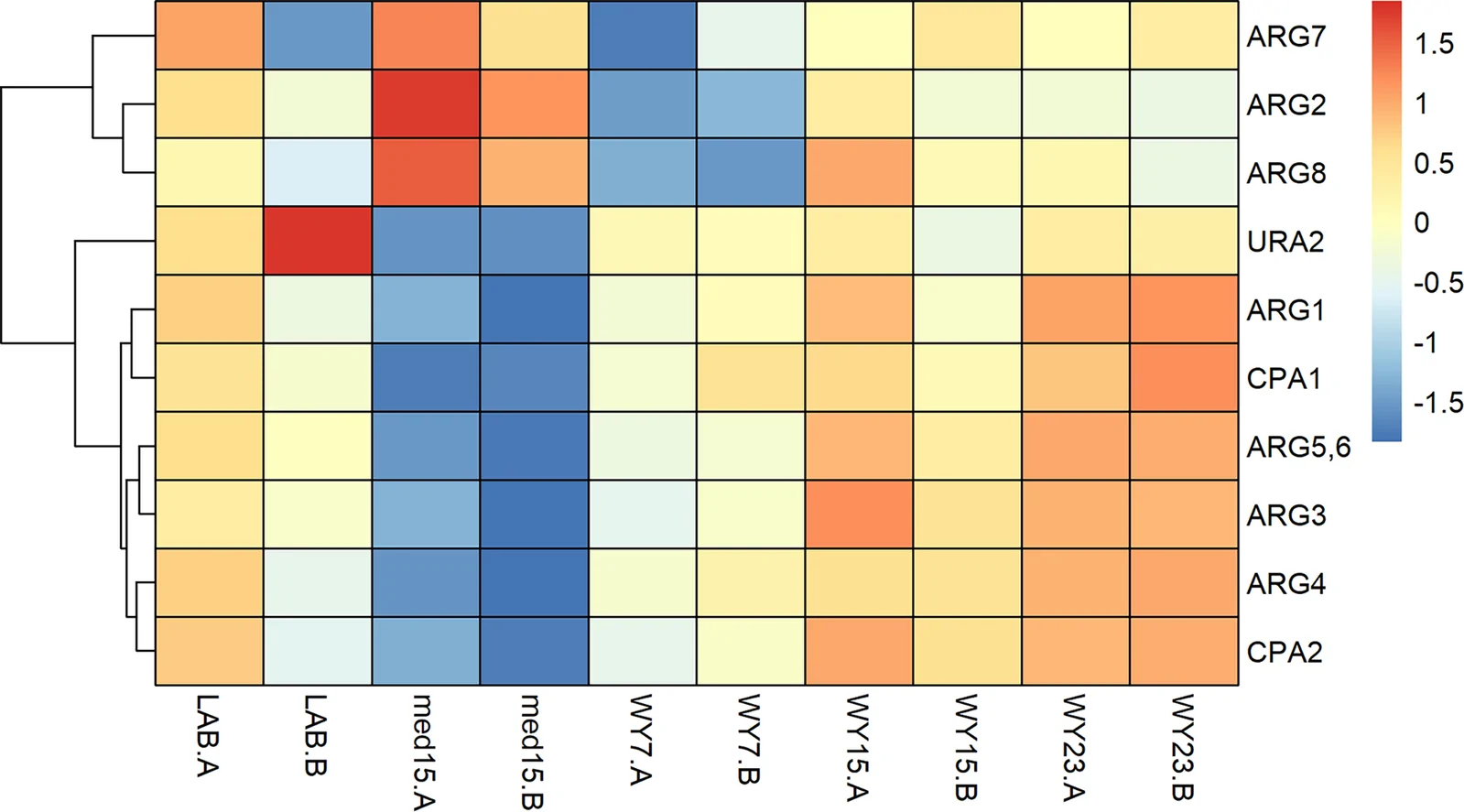
Heatmap representation of expression levels (Bowtie2) scaled per row from log2 transformed counts, in strains with different *MED15* alleles for various genes in the arginine biosynthesis pathway as defined by the BioCyc database [54]. Two biological replicates (**a, b**) of each strain type are shown. Seven of the ten gene rows show relative downregulation in the *med15* mutant, pointing to their normal activation by Med15, whereas *ARG7*, *ARG2* and *ARG8* are more highly expressed in the mutant, suggesting repression by Med15. Strikingly, these genes are under-expressed in strains with the *MED15*_WY7_ allele. Likewise, there is modest upregulation of *ARG1 – CPA2* (bottom six rows) relative to *ARG2*, *ARG7* and *ARG8* in strains with the *MED15*_WY23_ allele.

Transcriptomes of strains with integrated *MED15*_WY7_ or *MED15*_WY15_ alleles were very similar to the *MED15*_LAB_ transcriptome at the log2 fold-change and significance thresholds shown in Fig. 6. To achieve additional resolution, we combined GO term enrichment and pathway enrichment analyses using slightly relaxed parameters (Bowtie2-DESeq2 data). We also evaluated overlap among the enriched pathways in the different *MED15*_WY_ strains (compared to *MED15*_LAB_) (Fig. S3). The Venn diagram in Fig. 8 compares the pathways that are enriched in differentially expressed genes in strains differing only in their *MED15* allele (*MED15*_WY23_, *MED15*_WY7_ and *MED15*_WY15_ and *med15*Δ) each compared to *MED15*_LAB_. We find, for example, that the reduced expression of genes in the arginine pathway that we observed in the *med15* deletion strain relative to lab (Figs 4 and 8, light blue) is mirrored by the elevated expression of many of the same genes in the comparison of *MED15*_WY23_ and *MED15*_LAB_ (Fig. 8, purple). This effect can also be seen in the elevated z-scores for *ARG1*, *ARG3, ARG4, ARG5, ARG6, CPA1* and *CPA2* in the *MED15*_WY23_ columns of the heatmap in Fig. 9. While the arginine pathway was not enriched in the *MED15*_WY7_ strain, there was a notable decrease in the z-scores for *ARG2*, *ARG7* and *ARG8* expression relative to *MED15*_LAB_, in contrast to the higher expression of this specific subset of arginine metabolism genes in the *med15* deletion strain (Fig. 9).

Similar overlap in pathways affected in the *med15*Δ mutant (reduced) and in strains with *MED15*_WY7_ alleles (elevated) are seen for genes involved in DNA metabolism including meiosis, cell cycle, replication and repair (Fig. 8, intersection of the blue and yellow regions). Likewise, an effect on branched chain amino acid biosynthesis is seen in the *med15*Δ mutant, *MED15*_WY7_, and *MED15*_WY23_ (Fig. 8, intersection of the blue, yellow and purple regions).

Some affected pathways in strains with *MED15*_WY_ alleles are only minimally affected in the *med15Δ* mutant. For example, *MED15_WY7_* and *MED15_WY15_* share a set of affected translation functions, ranging from translation initiation to nonsense-mediated decay and co-translational membrane targeting, that are not prominent among genes affected in the *med15Δ* mutant. Likewise, the effects of *MED15_WY23_* and *MED15_WY7_* compared with *MED15*_LAB_ on lysine biosynthesis, and of *MED15_WY23_* and *MED15_WY15_* compared with *MED15*_LAB_ on aerobic respiration and the urea cycle are not seen in the *med15Δ* mutant (Fig. 8)

### *MED15*-dependent arginine regulation affects fermentation efficiency

Given the pronounced effect of *MED15_WY23_* on arginine biosynthetic genes, we investigated the relevance of the arginine biosynthetic pathway in WGJ fermentation. Fermentation experiments were first conducted in minimal synthetic dextrose medium supplemented with 20% glucose and amino acids required by the strains. This media was then further supplemented with increasing amounts of arginine. As expected, we found that arginine supplementation (to 800 µg ml^−1^) reliably reduced the time to 50% weight loss by ~9%, and that this reduction took place regardless of *MED15* genotype (Fig. S4) [67].

**Fig. 10.**
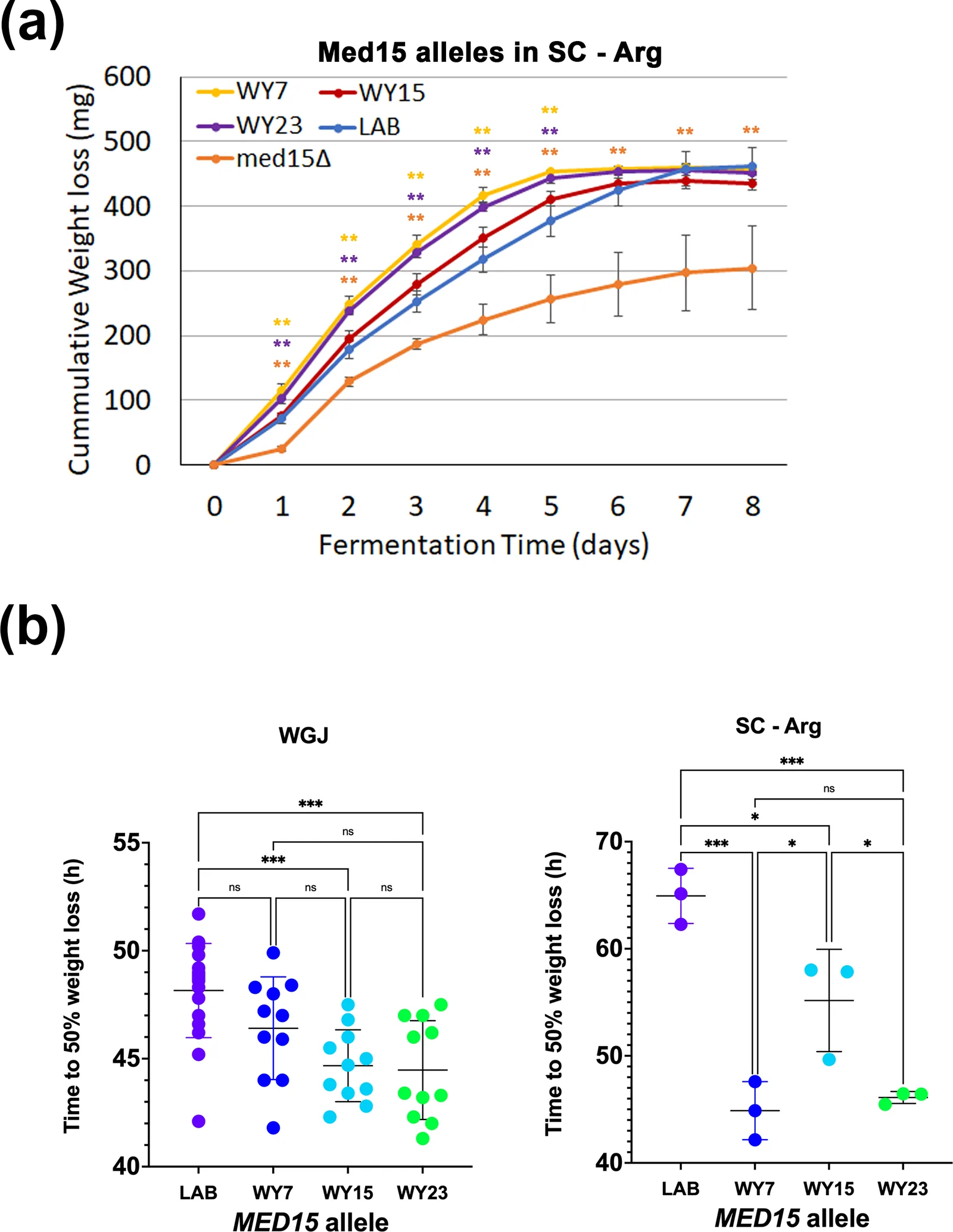
Improved fermentation among strains with specific *MED15*_WY_ alleles is evident in media lacking arginine. (**a**) Fermentation curves in SC media without arginine (containing all other amino acids) across strains with different *MED15* alleles carried episomally. Significant differences between each strain and *MED15*_LAB_ determined using two-tailed t-tests, **P<0.01. (**b**) Comparison of the fermentation efficiency of strains with different *MED15*_WY_ alleles in WGJ and SC-Arg. Left, time to 50% weight loss for strains with different *MED15*_WY_ alleles in WGJ (data from Table 5 in Cooper *et al*. [1]). Right, time to 50% wt loss for strains with different integrated *MED15*_WY_ alleles in SC media with 20% glucose lacking arginine. Individual data points are plotted with mean and sd shown as horizontal lines. Significance was determined with one-way ANOVA and Tukey post-hoc tests, *P<0.05, **P<0.005, ****P<0.0001.

The impact of arginine limitation was evaluated by conducting fermentation in SC media with 20% glucose and all amino acids except for arginine (SC-Arg) (Fig. 10). We hypothesized that *MED15*_WY_ alleles might tolerate the absence of arginine better than strains with the *MED15*_LAB_ allele due to alterations in the expression of amino acid metabolic genes and better utilization of available nitrogen sources [1, 68]. The time to 50% weight loss in the two types of media (WGJ and SC-Arg) for each strain is given in Fig. 10b (data for WGJ is from Table 5 in Cooper *et al*. [1]; and was conducted on strains with episomal *MED15* alleles). Interestingly, there are no significant distinctions among strains with different *MED15*_WY_ alleles in WGJ. However, strains with the *MED15*_WY7_ and *MED15*_WY23_ alleles clearly outperformed strains with the *MED15*_LAB_ and the *MED15*_WY15_ allele in media lacking arginine. This is consistent with the observation that arginine biosynthetic genes are elevated in the *MED15*_WY23_ strain during fermentation.

## Discussion

Previously, we found that *med15Δ* yeast strains were compromised in the efficiency of the fermentation process, while *MED15* alleles isolated from WY strains and swapped into a lab strain background in place of the *MED15*_LAB_ allele conferred a modest enhancement of fermentation [1]. It is striking that a single gene from a WY strain can measurably alter fermentation when swapped into a standard laboratory strain. Yeast fermentation is a complex physiological process involving hundreds of genes that regulate metabolism, stress tolerance and nutrient uptake among other processes. The change in fermentation due to a single genetic change emphasizes that Med15 occupies a key regulatory control point for fermentation producing observable physiological differences. This effect motivated the current follow-up transcriptomic study to discern what new patterns of *MED15*_WY_-dependent gene expression might account for the fermentation booster phenotype.

### Transcriptional patterns in YPD are consistent with a role for *MED15* in ethanol production

Our analysis of the role of *MED15* in fermentation was conducted in three parts. We first examined the effect of the *MED15*_LAB_ allele on gene expression of specific fermentation-related genes in standard media YPD with 2% glucose (qRT-PCR, Fig. 1). Second, we looked at the effect of *MED15* more globally in RNA-Seq experiments during WGJ-based alcoholic fermentation (RNA-Seq, Figs 3, 4 and 5). And third, we compared the transcriptomes of strains with *MED15*_LAB_ and *MED15*_WY_ alleles.

Our targeted qRT-PCR data (Fig. 1b) showed that *MED15* plays a broad role in regulating levels of metabolic enzymes in many steps in the glycolytic and alcoholic fermentation pathways. The expression patterns suggest that *MED15* normally functions to promote ethanol production (Fig. 1, see *ADH1* and *ADH2*). Our expression data also indicate that *MED15* both positively and negatively regulates paralogous or redundant genes in multiple steps of these pathways during log-phase growth in YPD. For example, *MED15* positively regulates the major isoform *PDC1* and downregulates the minor isoform *PDC6. MED15* positively regulates *ADH1,* which converts acetaldehyde to ethanol, and negatively regulates *ADH2,* which converts ethanol back to acetate [17]. Deletion of *ADH2* has been explored for strain engineering to increase the production of bioethanol [69]. In quadruple mutants where the only ADH maintained is *ADH1*, yeast still produce wild-type levels of ethanol [70]. Therefore, considering the sum of *MED15*-dependent regulation of *ADH* genes, metabolism is expected to be skewed towards ethanol production.

In addition to ethanol metabolism and fermentation genes, we selected genes based on their reported importance during fermentation physiology and metabolite production that influence flavour and stress adaptation [59–61, 71]. For example, glycerol, produced by the *GPD1* gene, contributes to mouthfeel, sweetness and smoothness in wine and is beneficial at the start of fermentation for osmoregulation given the high sugar content of the grape or WGJ [6, 72]. Yeast strains have also been engineered to produce less ethanol by the overexpression of *GPD1* to divert carbon to glycerol [61]. Finally, we found Med15 regulates amino acid metabolic genes, including *ATF2*, *ARO10*, *MET10*, and to a lesser degree, *IRC7* during log-phase growth in YPD (Fig. 1b), which influence volatile compound profiles and sulfite production among others [73–76].

While winemakers and producers of bioethanol seek to engineer strains for ethanol production, it is possible that natural variation in the general transcriptional regulator *MED15* could have permitted evolution to accomplish that same task.

### Transcriptional patterns under WGJ fermentation conditions

The *MED15* transcriptome is highly specialized during fermentation of WGJ. Less than 1% of DRGs in WGJ (20% glucose) are also identified in YPD (2% glucose). Our RNA-Seq analysis confirmed positive regulation by Med15 of many glycolytic and alcoholic fermentation genes (Figs 3 and 4). The regulation of these metabolic genes is an obvious explanation for the fermentation deficiencies we previously described for the *med15Δ* strain [1]. In addition to genes involved in the metabolism of glucose, *MED15* regulates a variety of disparate cellular activities during fermentation including cell wall homeostasis, cell cycle, sexual reproduction, carbohydrate transport and amino acid biosynthesis (Figs 3 and 8). The regulation of these other cellular activities may also contribute to the fermentation phenotype of the *med15Δ* strain. For example, the yeast cell wall is known to change in composition depending on growth phase and the composition of the media [77, 78]. Specifically, growth in nitrogen-limiting media results in cell wall deformations potentially due to a reduced number of linkages between cell wall subunits [79].

Subtelomeric genes are silenced through heterochromatinization in a process referred to as the telomere position effect (TPE) [80]. Our analysis of subtelomeric gene expression during WGJ fermentation indicates that *MED15* plays an enhanced role in telomeric silencing with 62% of analysed subtelomeric genes having significantly lower expression in the WT strain compared to the *med15Δ* strain. Med15-dependent subtelomeric silencing has been previously reported [62, 63, 81] and is observed to an extent on cells grown in minimal media laboratory conditions (SC); however, the effect on cells grown under rich media laboratory conditions (YPD) is modest and in opposition to the effect during WGJ fermentation (Fig. 5b). This suggests that either *MED15*-dependent telomeric silencing is induced in grape juice fermentation, and other nutrient-limited media, but not YPD, or that *MED15*-independent silencing is present in YPD but not grape juice fermentation. During fermentation, reinforcement of TPE may conserve cellular energy by limiting transcription of non-essential genes and stabilizing genome structure under stress.

The mechanism of enhanced subtelomeric gene silencing observed during fermentation may be related to the increased levels of H4K16Ac and reduced levels of Sir2 binding at telomeres in Mediator tail subunit deletions [81]. Sir3 occupancy at telomeres is decreased in a *med15Δ* strain, which would correspond with a reduction in Sir2 binding [62, 63]. Another potential mechanism by which Med15 could influence telomeric silencing during fermentation is through its known transcription factor interacting partners. At least two Med15 interacting TFs have a secondary role in silencing of subtelomeric genes. Oaf1 and Msn4 exert this effect by conditionally binding to telomeric proto-silencing regions known as X-elements without their usual co-activating protein partners, usually in a condition-specific manner. The negative regulatory function of Oaf1 corresponds to Oaf1 conditionally binding X elements at telomeres while growing in media with oleate [82]. The WGJ environment may correspond to stabilization or increased binding of TFs to X-elements to promote silencing in a *MED15*-dependent manner. The reduced telomeric silencing in the deletion strain may be due to low levels of fatty acid production (a known phenotype of *med15* mutants [83–85]) and thus less Oaf1 binding to X-elements.

### *MED15*_WY23_ affects expression of metabolic genes including the arginine pathway during fermentation

We found previously that some *MED15* alleles from WY confer enhanced fermentation traits when substituted for the *MED15*_LAB_ allele in lab strains [1]. We therefore analysed expression patterns in lab strains expressing one of two *MED15* alleles (*MED15*_WY7_ and *MED15*_WY15_) belonging to the European wine clade and a *MED15* allele from a palm WY (*MED15*_WY23_). A set of genes differentially regulated by WY alleles were identified by GO and pathway enrichment analyses (Figs 6–8). *MED15*_WY23_ had the largest impact on the transcriptome. This allele differs from the *MED15*_LAB_ allele primarily in the length of poly-Q tracts, there being only three non-synonymous substitutions outside of poly-Q tracts, and no impact of removing them was observed in our previous studies [1]. Hence, the difference in Q tract lengths in the *MED15*_WY23_ allele gene is likely accountable for the new transcriptome. The differentially expressed genes in strains with *MED15*_WY23_ compared to *MED15*_LAB_ were enriched in metabolic activities as well as stress response, detoxification, transporter activity and mitochondrial/peroxisomal metabolism (Figs 6d and 7, Table S2).

Especially noteworthy was the elevated expression of many arginine biosynthesis pathway enzymes (*ARG1, 3–6*) (Figs 6d and 7) in the *MED15*_WY23_ strain. These same arginine biosynthesis pathway enzymes were all significantly affected in comparisons of the *MED15*_LAB_ strain compared to a *med15* deletion strain (Fig. 4). This leads us to conclude that arginine biosynthesis from glutamine is dependent on *MED15* and that the activation of these genes is enhanced by the *MED15*_23_ allele.

In the row-normalized heatmap shown in Fig. 9, it was possible to detect not only the previously noted decrease in *ARG1* and *ARG3-6* expression in the *med15* deletion mutant but also coordinated decreases in *CPA1* and *CPA2* gene expression, whose contribution to arginine biosynthesis is shown in Fig. 4c, and in *URA2*, a bifunctional protein whose N terminus comprises a carbamoyl phosphate synthetase domain that converts ammonium to carbamoyl phosphate, which feeds back into the urea cycle. Also apparent in the deletion mutant is a relative increase in expression of the *ARG2*, *ARG7* and *ARG8* genes that support the conversion of glutamate to ornithine and feeds back into the arginine biosynthetic pathway via *ARG3*-dependent conversion to citrulline. The heatmap also reveals that the *MED1*5_WY23_ allele is not the only WY allele affecting the relative expression of genes in the arginine biosynthetic pathway. Strains with *MED15*_WY7_ show a relative reduction in the same *ARG2*, *ARG7, ARG8* gene cluster that is more abundantly expressed in the deletion strain. These patterns suggest that the opposing expression of the two clusters of arginine biosynthetic genes is an important contributor to alcoholic fermentation and that small *MED15*-dependent adjustments that exaggerate the differential between the two clusters are one mechanism by which fermentation characteristics of a strain can be improved.

Arginine is one of the major amino acids found in grape must and an important nitrogen source for yeast [68, 86]. Arginine is cleaved by arginase (*CAR1* gene) into ornithine and urea, both of which can be used as a nitrogen source. Urea can be further catabolized to NH_3_ (ammonia) and CO_2_. This is an energy-dependent process carried out by *DUR12* encoding urea amidolyase. Arginine and urea are considered secondary nitrogen sources used by the cell only when better sources like ammonia and glutamine are no longer present in the growth media. Constitutive expression of *DUR12* in WY improves the degradation of urea during grape must fermentation, a reduction in fermentation time and increased ethanol production [87, 88]. Arginine is also known to have a protective effect against ethanol stress, in part by playing a role in the maintenance of cell wall integrity and in reducing oxidative stress [89], so that elevated arginine levels in strains with *MED15*_WY_ alleles may also contribute to the ability of yeast strains to survive the stressful environment resulting from WGJ fermentation.

### Arginine biosynthesis involvement in *MED15*-dependent fermentation phenotypes

The impact of the *MED15* allele was clear in fermentation reactions conducted in media lacking arginine. Strains with the *MED15*_WY7_ or *MED15*_WY23_ allele performed significantly better than strains with the *MED15*_LAB_ allele in arginine-deficient media, while strains with the *MED15*_WY15_ allele were unaffected (Fig. 10). Based on the heatmap in Fig. 9, we hypothesize that the *MED15*_WY7_ and *MED15*_WY23_ transcriptomes optimize arginine levels using two strategies. *MED15*_WY23_ promotes arginine production from glutamine (*CPA1*, *CPA2*, *ARG1, 3, 4*), while *MED15*_WY7_ does so by limiting ornithine production (Figs 7 and 9).

Although *MED15*_WY7_ and *MED15*_WY15_ alleles had transcriptomes nearly identical to *MED15*_LAB_ at the tested time point, there were a small number of differentially expressed genes, and these overlapped with differentially expressed genes in the *MED15*_WY23_ analysis, including *MCH5*, *SNQ2*, and *LYS20*. Since these genes are differentially regulated by at least two of the tested *MED15*_WY_ alleles, they are also candidate contributors to WGJ fermentation efficiency. *MCH5*, a riboflavin transport gene [90] was previously identified as a fermentation stress response gene induced early in fermentation [91]. In addition to riboflavin in the environment, *MCH5* is regulated by proline [92], which may be relevant to the limited nitrogen environment of WGJ. *LYS20* encodes a homocitrate synthase and may be beneficial in the limited nitrogen environment of WGJ. Additionally, *LYS20* functions in DNA repair [93], which may also be beneficial in the harsh WGJ environment. *SNQ2* is a transporter involved in pleiotropic drug resistance, regulated by Pdr1/3 [94], and has previously been shown to serve a protective role in the fermentation environment [95]. In preliminary experiments, we found that deletion of the *SNQ2* gene in strains with the *MED15_WY7_* allele exhibited reduced fermentation (data not shown).

Analysis of pathways rather than GO enrichment terms led to additional hypotheses concerning gene expression patterns that may contribute to increased fermentation rates as summarized in Fig. 8. Roles for general amino acid biosynthesis, translation, DNA metabolism and mitochondrial dynamics in fine-tuning fermentation await further analysis.

One limitation of this study is that the influence of the *MED15*_WY_ alleles on gene expression may vary at different times during the fermentation process. Future transcriptomic analyses of these and additional strains throughout the time course of a fermentation reaction could reveal the full effect of *MED15* alleles on gene expression responsible for the boosted fermentation phenotypes we observed [1].

## Supplementary material

10.1099/mgen.0.001811 Supplementary Material 1.

## Abbreviations

ACS: acetyl CoA synthetase
ADH: alcohol dehydrogenase
ALD: aldehyde dehydrogenase
°Bx: degrees Brix
CP: crossing point
dNTP: deoxynucleotide triphosphate
DRG: differentially regulated gene
ESR: environmental stress response
5-FOA: 5-fluoroorotic acid
GO: gene ontology
IIHG: Iowa Institute for Human Genetics
ITS: interspersed telomeric repeats
MCHM: 4-methylcyclohexane methanol
NADH: nicotinamide adenine dinucleotide + hydrogen
PDC: pyruvate decarboxylase
poly-Q: polyglutamine
RT: room temperature
SC/SC-AA: synthetic complete medium lacking specified amino acid(s)
TPE: telomere position effect
WGJ: white grape juice
WY: wine yeast
YPD: yeast peptone dextrose

## References

[1] Cooper DG, Jiang Y, Skuodas S, Wang L, Fassler JS (2021). Possible role for allelic variation in yeast *MED15* in ecological adaptation. Front Microbiol.

[2] Cooper DG, Fassler JS (2019). Med15: glutamine-rich mediator subunit with potential for plasticity. Trends Biochem Sci.

[3] Marsit S, Dequin S (2015). Diversity and adaptive evolution of *Saccharomyces* wine yeast: a review. FEMS Yeast Res.

[4] Mortimer RK (2000). Evolution and variation of the yeast (*Saccharomyces*) genome. Genome Res.

[5] Fleet GH (1993). Wine Microbiology and Biotechnology.

[6] Pretorius IS (2000). Tailoring wine yeast for the new millennium: novel approaches to the ancient art of winemaking. Yeast.

[7] Varela C, Torrea D, Schmidt SA, Ancin-Azpilicueta C, Henschke PA (2012). Effect of oxygen and lipid supplementation on the volatile composition of chemically defined medium and Chardonnay wine fermented with *Saccharomyces cerevisiae*. Food Chem.

[8] Peltier E, Bernard M, Trujillo M, Prodhomme D, Barbe J-C (2018). Wine yeast phenomics: a standardized fermentation method for assessing quantitative traits of *Saccharomyces cerevisiae* strains in enological conditions. PLoS One.

[9] Gallone B, Steensels J, Prahl T, Soriaga L, Saels V (2016). Domestication and divergence of *Saccharomyces cerevisiae* beer yeasts. Cell.

[10] Mendes-Ferreira A, del Olmo M, García-Martínez J, Jiménez-Martí E, Leão C (2007). *Saccharomyces cerevisiae* signature genes for predicting nitrogen deficiency during alcoholic fermentation. Appl Environ Microbiol.

[11] Mendes-Ferreira A, del Olmo M, García-Martínez J, Jiménez-Martí E, Mendes-Faia A (2007). Transcriptional response of *Saccharomyces cerevisiae* to different nitrogen concentrations during alcoholic fermentation. Appl Environ Microbiol.

[12] Legras J-L, Galeote V, Bigey F, Camarasa C, Marsit S (2018). Adaptation of *S. cerevisiae* to fermented food environments reveals remarkable genome plasticity and the footprints of domestication. Mol Biol Evol.

[13] Johnston JR, Baccari C, Mortimer RK (2000). Genotypic characterization of strains of commercial wine yeastsby tetrad analysis. Res Microbiol.

[14] Fay JC, Benavides JA (2005). Evidence for domesticated and wild populations of *Saccharomyces cerevisiae*. PLoS Genet.

[15] Crabtree HG (1929). Observations on the carbohydrate metabolism of tumours. Biochem J.

[16] De Deken RH (1966). The Crabtree effect: a regulatory system in yeast. J Gen Microbiol.

[17] de Smidt O, du Preez JC, Albertyn J (2008). The alcohol dehydrogenases of *Saccharomyces cerevisiae*: a comprehensive review. FEMS Yeast Res.

[18] Wills C (1976). Production of yeast alcohol dehydrogenase isoenzymes by selection. Nature.

[19] Ihmels J, Bergmann S, Gerami-Nejad M, Yanai I, McClellan M (2005). Rewiring of the yeast transcriptional network through the evolution of motif usage. Science.

[20] Myers LC, Gustafsson CM, Bushnell DA, Lui M, Erdjument-Bromage H (1998). The Med proteins of yeast and their function through the RNA polymerase II carboxy-terminal domain. Genes Dev.

[21] Dotson MR, Yuan CX, Roeder RG, Myers LC, Gustafsson CM (2000). Structural organization of yeast and mammalian mediator complexes. Proc Natl Acad Sci USA.

[22] Boube M, Joulia L, Cribbs DL, Bourbon HM (2002). Evidence for a mediator of RNA polymerase II transcriptional regulation conserved from yeast to man. Cell.

[23] Zhang F, Sumibcay L, Hinnebusch AG, Swanson MJ (2004). A triad of subunits from the Gal11/tail domain of Srb Mediator is an *in vivo* target of transcriptional activator Gcn4p. Mol Cell Biol.

[24] Gallagher JEG, Ser SL, Ayers MC, Nassif C, Pupo A (2020). The polymorphic PolyQ tail protein of the Mediator complex, Med15, regulates the variable response to diverse stresses. Int J Mol Sci.

[25] Cooper DG, Liu S, Grunkemeyer E, Fassler JS (2025). The role of Med15 sequence features in transcription factor interactions. Mol Cell Biol.

[26] Gemayel R, Chavali S, Pougach K, Legendre M, Zhu B (2015). Variable glutamine-rich repeats modulate transcription factor activity. Mol Cell.

[27] Bryan AC, Zhang J, Guo J, Ranjan P, Singan V (2018). A variable polyglutamine repeat affects subcellular localization and regulatory activity of a *Populus* ANGUSTIFOLIA protein. G3.

[28] O’Malley KG, Ford MJ, Hard JJ (2010). Clock polymorphism in Pacific salmon: evidence for variable selection along a latitudinal gradient. Proc Biol Sci.

[29] Caprioli M, Ambrosini R, Boncoraglio G, Gatti E, Romano A (2012). Clock gene variation is associated with breeding phenology and maybe under directional selection in the migratory barn swallow. PLoS One.

[30] Mortimer RK, Johnston JR (1986). Genealogy of principal strains of the yeast genetic stock center. Genetics.

[31] Brachmann CB, Davies A, Cost GJ, Caputo E, Li J (1998). Designer deletion strains derived from *Saccharomyces cerevisiae* S288C: a useful set of strains and plasmids for PCR-mediated gene disruption and other applications. Yeast.

[32] Bradbury JE, Richards KD, Niederer HA, Lee SA, Rod Dunbar P (2006). A homozygous diploid subset of commercial wine yeast strains. Antonie van Leeuwenhoek.

[33] Kim DH, Kim GS, Yun CH, Lee YC (2008). Functional conservation of the glutamine-rich domains of yeast Gal11 and human SRC-1 in the transactivation of glucocorticoid receptor Tau 1 in *Saccharomyces cerevisiae*. Mol Cell Biol.

[34] Ito H, Fukuda Y, Murata K, Kimura A (1983). Transformation of intact yeast cells treated with alkali cations. J Bacteriol.

[35] Gietz RD, Woods RA (2002). Transformation of yeast by lithium acetate/single-stranded carrier DNA/polyethylene glycol method. Meth Enzymol.

[36] Hu Z, Killion PJ, Iyer VR (2007). Genetic reconstruction of a functional transcriptional regulatory network. Nat Genet.

[37] Kemmeren P, Sameith K, van de Pasch LAL, Benschop JJ, Lenstra TL (2014). Large-scale genetic perturbations reveal regulatory networks and an abundance of gene-specific repressors. Cell.

[38] Reimand J, Vaquerizas JM, Todd AE, Vilo J, Luscombe NM (2010). Comprehensive reanalysis of transcription factor knockout expression data in *Saccharomyces cerevisiae* reveals many new targets. Nucleic Acids Res.

[39] Giaever G, Chu AM, Ni L, Connelly C, Riles L (2002). Functional profiling of the *Saccharomyces cerevisiae* genome. Nature.

[40] Collart MA, Oliviero S (2001). Preparation of yeast RNA. Curr Protoc Mol Biol.

[41] Jalili V, Afgan E, Gu Q, Clements D, Blankenberg D (2020). The Galaxy platform for accessible, reproducible and collaborative biomedical analyses: 2020 update. Nucleic Acids Res.

[42] Langmead B, Salzberg SL (2012). Fast gapped-read alignment with Bowtie 2. Nat Methods.

[43] Liao Y, Smyth GK, Shi W (2014). FeatureCounts: an efficient general purpose program for assigning sequence reads to genomic features. Bioinformatics.

[44] Love MI, Huber W, Anders S (2014). Moderated estimation of fold change and dispersion for RNA-seq data with DESeq2. Genome Biol.

[45] Bray NL, Pimentel H, Melsted P, Pachter L (2016). Near-optimal probabilistic RNA-seq quantification. Nat Biotechnol.

[46] Ritchie ME, Phipson B, Wu D, Hu Y, Law CW (2015). limma powers differential expression analyses for RNA-sequencing and microarray studies. Nucleic Acids Res.

[47] Pimentel H, Bray NL, Puente S, Melsted P, Pachter L (2017). Differential analysis of RNA-seq incorporating quantification uncertainty. Nat Methods.

[48] Law CW, Chen Y, Shi W, Smyth GK (2014). voom: Precision weights unlock linear model analysis tools for RNA-seq read counts. Genome Biol.

[49] Soneson C, Love MI, Robinson MD (2015). Differential analyses for RNA-seq: transcript-level estimates improve gene-level inferences. F1000Res.

[50] Durinck S, Spellman PT, Birney E, Huber W (2009). Mapping identifiers for the integration of genomic datasets with the R/Bioconductor package biomaRt. *Nat Protoc*.

[51] Goedhart J, Luijsterburg MS (2020). VolcaNoseR is a web app for creating, exploring, labeling and sharing volcano plots. Sci Rep.

[52] Chen H, Boutros PC (2011). VennDiagram: a package for the generation of highly-customizable Venn and Euler diagrams in R. BMC Bioinformatics.

[53] Bult CJ, Sternberg PW (2023). The alliance of genome resources: transforming comparative genomics. Mamm Genome.

[54] Karp PD, Billington R, Caspi R, Fulcher CA, Latendresse M (2019). The BioCyc collection of microbial genomes and metabolic pathways. Brief Bioinform.

[55] Korotkevich G, Sukhov V, Budin N, Shpak B, Artyomov MN (2021). Fast gene set enrichment analysis. *Bioinformatics*.

[56] Kamburov A, Herwig R (2022). ConsensusPathDB 2022: molecular interactions update as a resource for network biology. Nucleic Acids Res.

[57] Kolde R (2025). pheatmap.

[58] Ansari SA, Ganapathi M, Benschop JJ, Holstege FCP, Wade JT (2012). Distinct role of Mediator tail module in regulation of SAGA-dependent, TATA-containing genes in yeast. EMBO J.

[59] Rossignol T, Dulau L, Julien A, Blondin B (2003). Genome-wide monitoring of wine yeast gene expression during alcoholic fermentation. Yeast.

[60] Holt S, Miks MH, de Carvalho BT, Foulquié-Moreno MR, Thevelein JM (2019). The molecular biology of fruity and floral aromas in beer and other alcoholic beverages. FEMS Microbiol Rev.

[61] Pretorius I (2016). Conducting wine symphonics with the aid of yeast genomics. *Beverages*.

[62] Lenstra TL, Benschop JJ, Kim T, Schulze JM, Brabers NACH (2011). The specificity and topology of chromatin interaction pathways in yeast. Mol Cell.

[63] Hocher A, Ruault M, Kaferle P, Descrimes M, Garnier M (2018). Expanding heterochromatin reveals discrete subtelomeric domains delimited by chromatin landscape transitions. Genome Res.

[64] Naumov G, Turakainen H, Naumova E, Aho S, Korhola M (1990). A new family of polymorphic genes in *Saccharomyces cerevisiae*: α-galactosidase genes MEL1-MEL7. Molec Gen Genet.

[65] Chow THC, Sollitti P, Marmur J (1989). Structure of the multigene family of MAL loci in *Saccharomyces*. *Mol Gen Genet*.

[66] Charron MJ, Read E, Haut SR, Michels CA (1989). Molecular evolution of the telomere-associated MAL loci of *Saccharomyces*. Genetics.

[67] Gutiérrez A, Chiva R, Guillamón JM (2015). Arginine addition in the stationary phase influences the fermentation rate and synthesis of aroma compounds in a synthetic must fermented by three commercial wine strains. *Food Sci Technol*.

[68] Jiranek V, Langridge P, Henschke PA (1995). Amino acid and ammonium utilization by *Saccharomyces cerevisiae* wine yeasts from a chemically defined medium. Am J Enol Vitic.

[69] Xue T, Liu K, Chen D, Yuan X, Fang J (2018). Improved bioethanol production using CRISPR/Cas9 to disrupt the ADH2 gene in *Saccharomyces cerevisiae*. *World J Microbiol Biotechnol*.

[70] de Smidt O, du Preez JC, Albertyn J (2012). Molecular and physiological aspects of alcohol dehydrogenases in the ethanol metabolism of *Saccharomyces cerevisiae*. FEMS Yeast Res.

[71] Dubin RA, Needleman RB, Gossett D, Michels CA (1985). Identification of the structural gene encoding maltase within the MAL6 locus of *Saccharomyces carlsbergensis*. J Bacteriol.

[72] Tamás MJ, Luyten K, Sutherland FC, Hernandez A, Albertyn J (1999). Fps1p controls the accumulation and release of the compatible solute glycerol in yeast osmoregulation. Mol Microbiol.

[73] Deed RC, Hou R, Kinzurik MI, Gardner RC, Fedrizzi B (2019). The role of yeast ARO8, ARO9 and ARO10 genes in the biosynthesis of 3-(methylthio)-1-propanol from L-methionine during fermentation in synthetic grape medium. FEMS Yeast Res.

[74] Linderholm A, Dietzel K, Hirst M, Bisson LF (2010). Identification of MET10-932 and characterization as an allele reducing hydrogen sulfide formation in wine strains of *Saccharomyces cerevisiae*. Appl Environ Microbiol.

[75] Roncoroni M, Santiago M, Hooks DO, Moroney S, Harsch MJ (2011). The yeast IRC7 gene encodes a β-lyase responsible for production of the varietal thiol 4-mercapto-4-methylpentan-2-one in wine. Food Microbiol.

[76] Zhang J, Zhang C, Qi Y, Dai L, Ma H (2014). Acetate ester production by Chinese yellow rice wine yeast overexpressing the alcohol acetyltransferase-encoding gene ATF2. Genet Mol Res.

[77] Varelas V, Sotiropoulou E, Karambini X, Liouni M, Nerantzis E (2017). Impact of glucose concentration and NaCl osmotic stress on yeast cell wall β-d-glucan formation during anaerobic fermentation process. *Fermentation*.

[78] Kim KS, Yun HS (2006). Production of soluble β-glucan from the cell wall of *Saccharomyces cerevisiae*. Enzyme Microbial Technol.

[79] McMurrough I, Rose AH (1967). Effect of growth rate and substrate limitation on the composition and structure of the cell wall of *Saccharomyces cerevisiae*. Biochem J.

[80] Ottaviani A, Gilson E, Magdinier F (2008). Telomeric position effect: from the yeast paradigm to human pathologies?. Biochimie.

[81] Peng J, Zhou JQ (2012). The tail-module of yeast Mediator complex is required for telomere heterochromatin maintenance. Nucleic Acids Res.

[82] Smith JJ, Miller LR, Kreisberg R, Vazquez L, Wan Y (2011). Environment-responsive transcription factors bind subtelomeric elements and regulate gene silencing. Mol Syst Biol.

[83] Qi Y, Liu H, Yu J, Chen X, Liu L (2017). Med15B regulates acid stress response and tolerance in *Candida glabrata* by altering membrane lipid composition. Appl Environ Microbiol.

[84] Taubert S, Van Gilst MR, Hansen M, Yamamoto KR (2006). A Mediator subunit, MDT-15, integrates regulation of fatty acid metabolism by NHR-49-dependent and -independent pathways in *C. elegans*. Genes Dev.

[85] Thakur JK, Arthanari H, Yang F, Chau KH, Wagner G (2009). Mediator subunit Gal11p/MED15 is required for fatty acid-dependent gene activation by yeast transcription factor Oaf1p. J Biol Chem.

[86] Henschke PA, Jiranek V, Fleet GH (1993). Wine Microbiology and Biotechnology.

[87] Coulon J, Husnik JI, Inglis DL, van der Merwe GK, Lonvaud A (2006). Metabolic engineering of *Saccharomyces cerevisiae* to minimize the production of ethyl carbamate in wine. Am J Enol Vitic.

[88] Shalamitskiy MY, Tanashchuk TN, Cherviak SN, Vasyagin EA, Ravin NV (2023). Ethyl carbamate in fermented food products: sources of appearance, hazards and methods for reducing its content. Foods.

[89] Cheng Y, Du Z, Zhu H, Guo X, He X (2016). Protective effects of arginine on *Saccharomyces cerevisiae* against ethanol stress. Sci Rep.

[90] Reihl P, Stolz J (2005). The monocarboxylate transporter homolog Mch5p catalyzes riboflavin (vitamin B2) uptake in *Saccharomyces cerevisiae*. J Biol Chem.

[91] Marks VD, Ho Sui SJ, Erasmus D, van der Merwe GK, Brumm J (2008). Dynamics of the yeast transcriptome during wine fermentation reveals a novel fermentation stress response. FEMS Yeast Res.

[92] Spitzner A, Perzlmaier AF, Geillinger KE, Reihl P, Stolz J (2008). The proline-dependent transcription factor Put3 regulates the expression of the riboflavin transporter MCH5 in *Saccharomyces cerevisiae*. Genetics.

[93] Torres-Machorro AL, Aris JP, Pillus L (2015). A moonlighting metabolic protein influences repair at DNA double-stranded breaks. Nucleic Acids Res.

[94] Rogers B, Decottignies A, Kolaczkowski M, Carvajal E, Balzi E (2001). The pleitropic drug ABC transporters from *Saccharomyces cerevisiae*. J Mol Microbiol Biotechnol.

[95] Watanabe M, Mizoguchi H, Nishimura A (2000). Disruption of the ABC transporter genes PDR5, YOR1, and SNQ2, and their participation in improved fermentative activity of a sake yeast mutant showing pleiotropic drug resistance. J Biosci Bioengineer.

[96] Sikorski RS, Hieter P (1989). A system of shuttle vectors and yeast host strains designed for efficient manipulation of DNA in *Saccharomyces cerevisiae*. Genetics.

