# Supplementary material for: Transcriptomic shift in ethanol and amino acid metabolic genes regulated by Med15 during alcoholic fermentation
Source: Microb Genom. 2026 Sep 3;12(9):001811. doi: 10.1099/mgen.0.001811 (PMC13541290; doi:10.1099/mgen.0.001811)
Supplement: Supplementary file 1 [file mgen-12-01811-s001.pdf]

Transcriptomic shift in ethanol and amino acid metabolic genes regulated by Med15 during alcoholic fermentation

David G. Cooper[1,2], Emma Grunkemeyer[1] and Jan S. Fassler[1,3]

[1]Department of Biology, University of Iowa, Iowa City, IA

[2]Department of Pharmaceutical Sciences, Butler University, Indianapolis, IN

[3]Corresponding Author

Supplementary Figures and Tables

Figure S1 – RNA Sequencing Biological Replicate Comparison

Figure S2 –Comparison of Differentially Regulated Genes using Alternative Analytical Pipelines

Figure S3 – Pathway Enrichment Overlap between *MED15* strains

Figure S4 – Arginine Supplemented Fermentation

Table S1 – Genes with an average of 10,000 or more additional counts using kallisto vs. Bowtie2

Table S2 – Genes Differentially Regulated by *MED15*$_{WY23}$ During WGJ Fermentation

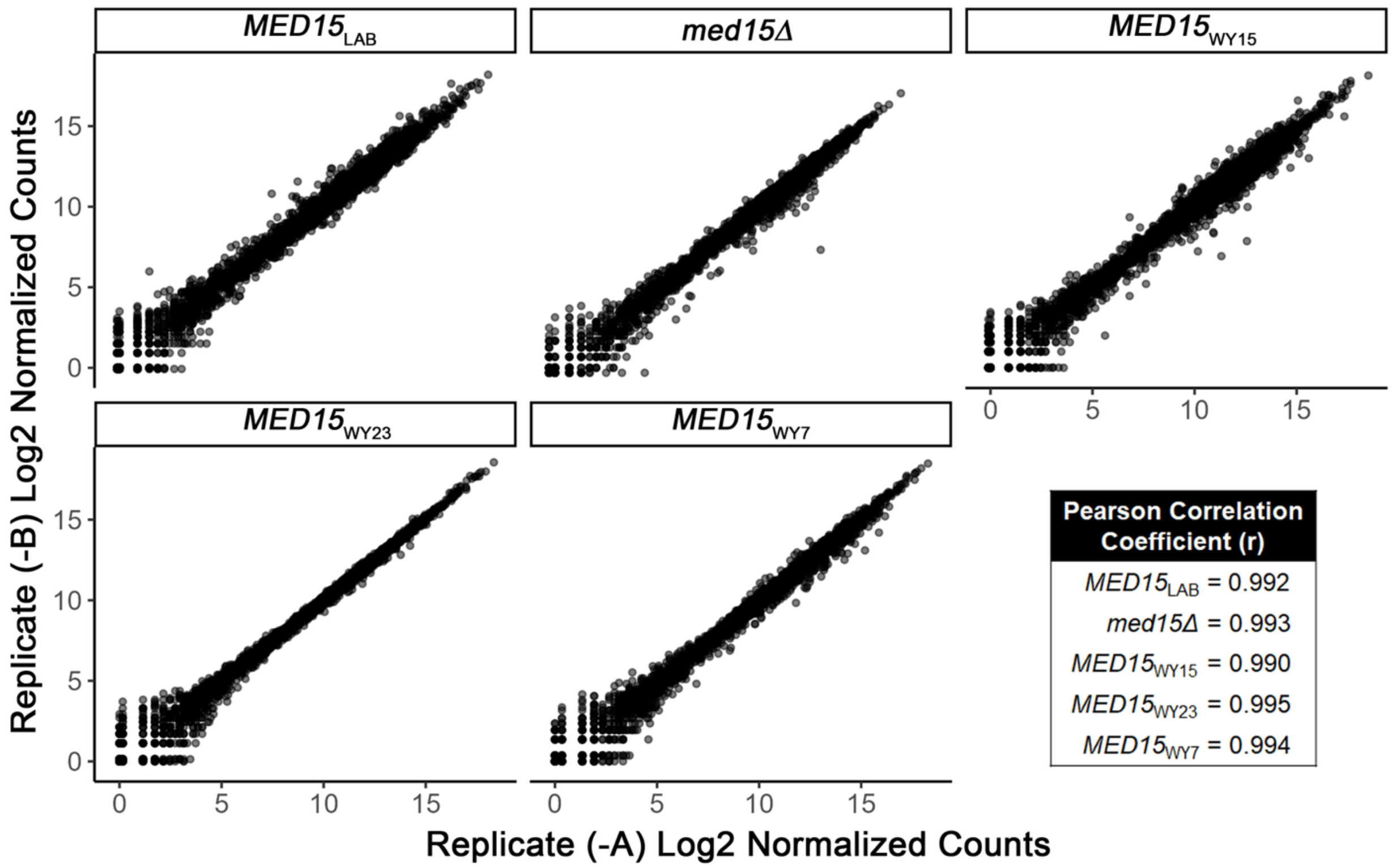

Figure S1. RNA Sequencing Biological Replicate Comparison

Correlation analysis between log2 transformed normalized counts of two biological replicates (-A replicate counts on x-axis and -B replicate counts on y-axis) for each *MED15* genotype. Pearson correlation coefficients (r) range between 0.990 and 0.995.

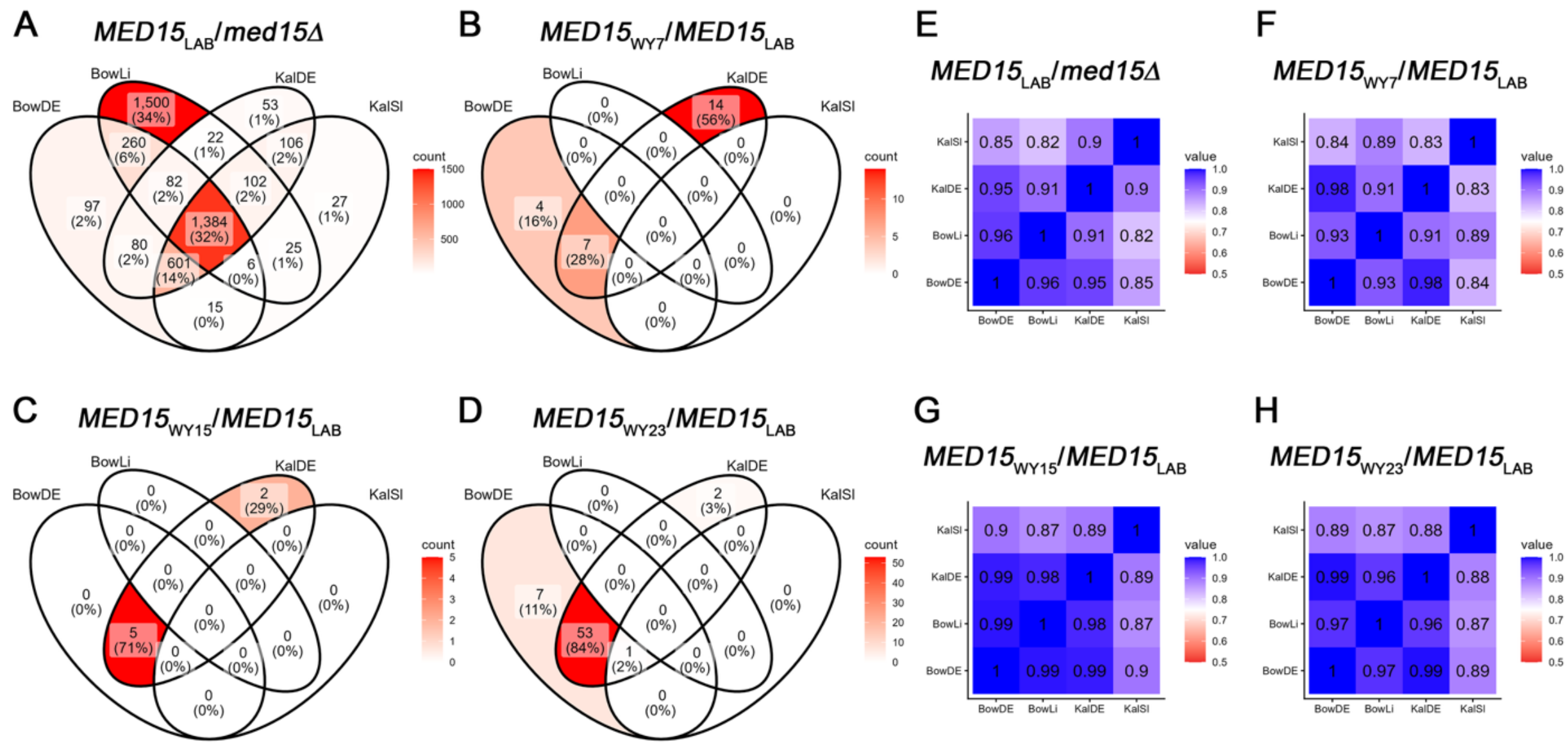

Figure S2. Comparison of Differentially Regulated Genes using Alternative Analytical Pipelines

(A-D) Venn diagrams showing overlap of genes with adjusted p-values < 0.05 for each of four methods (Bowtie2 -> DESeq2 (BowDE), Bowtie2 -> limma (BowLi), kallisto -> DESeq2 (KalDE), kallisto -> sleuth (KalSl)) for the four comparisons (LAB vs deletion, WY7 vs LAB, WY15 vs LAB, and WY23 vs LAB). **(E-H)** Pearson correlation matrixes of the statistical values for the shared set of genes for each of four methods for the four comparisons.

```
$`LAB:WY7:WY15`
[1] "Formation of a pool of free 40S subunits | Reactome"
[2] "GTP hydrolysis and joining of the 60S ribosomal subunit |
Reactome"

$LAB
 [1] "Basal transcription factors - Saccharomyces cerevisiae
(budding yeast) | KEGG"
 [2] "Estrogen-dependent gene expression | Reactome"
 [3] "Gene expression (Transcription) | Reactome"
 [4] "Glucose metabolism | Reactome"
 [5] "Glycolysis | Reactome"
 [6] "Metabolism of carbohydrates | Reactome"
 [7] "Orc1 removal from chromatin | Reactome"
 [8] "Pyruvate metabolism - Saccharomyces cerevisiae (budding
yeast) | KEGG"
 [9] "RNA Polymerase II Pre-transcription Events | Reactome"
[10] "RNA Polymerase II Promoter Escape | Reactome"
[11] "RNA Polymerase II Transcription Elongation | Reactome"
[12] "RNA Polymerase II Transcription Initiation And Promoter
Clearance | Reactome"
[13] "RNA Polymerase II Transcription Initiation | Reactome"
[14] "RNA Polymerase II Transcription Pre-Initiation And
Promoter Opening | Reactome"
[15] "RNA Polymerase II Transcription | Reactome"
[16] "RNA polymerase II transcribes snRNA genes | Reactome"
[17] "Sulfur metabolism - Saccharomyces cerevisiae (budding
yeast) | KEGG"
[18] "Switching of origins to a post-replicative state |
Reactome"
[19] "gluconeogenesis | YeastCyc"

$WY7
 [1] "Base excision repair - Saccharomyces cerevisiae (budding
yeast) | KEGG"
 [2] "Cysteine and methionine metabolism - Saccharomyces
cerevisiae (budding yeast) | KEGG"
 [3] "DNA Double-Strand Break Repair | Reactome"
 [4] "Glycine, serine and threonine metabolism - Saccharomyces
cerevisiae (budding yeast) | KEGG"
 [5] "Homologous recombination - Saccharomyces cerevisiae
(budding yeast) | KEGG"
 [6] "MAPK signaling pathway - yeast - Saccharomyces cerevisiae
(budding yeast) | KEGG"
 [7] "Metabolism | Reactome"
 [8] "Mismatch Repair | Reactome"
```

```
 [9] "Mismatch repair (MMR) directed by MSH2:MSH6 (MutSalpha) |
Reactome"
[10] "Phenylalanine, tyrosine and tryptophan biosynthesis -
Saccharomyces cerevisiae (budding yeast) | KEGG"
[11] "RNA Polymerase I Promoter Clearance | Reactome"
[12] "RNA Polymerase I Promoter Escape | Reactome"
[13] "RNA Polymerase I Transcription | Reactome"
[14] "SUMOylation of DNA damage response and repair proteins |
Reactome"
[15] "cysteine biosynthesis IV (fungi) | YeastCyc"
[16] "superpathway of phenylalanine, tyrosine and tryptophan
biosynthesis | YeastCyc"
[17] "superpathway of sulfur amino acid biosynthesis
(<i>Saccharomyces cerevisiae</i>) | YeastCyc"

$WY15
[1] "Metabolism of proteins | Reactome"
[2] "TCA cycle II (plants and fungi) | YeastCyc"

$WY23
[1] "L-lysine biosynthesis IV | YeastCyc"
[2] "Respiratory electron transport, ATP synthesis by
chemiosmotic coupling, and heat production by uncoupling
proteins. | Reactome"
[3] "aerobic respiration (cytochrome c) | YeastCyc"
[4] "aerobic respiration (linear view) | YeastCyc"
[5] "urea cycle | YeastCyc"

$`LAB:WY7`
 [1] "Activation of the pre-replicative complex | Reactome"
 [2] "Cell Cycle | Reactome"
 [3] "Cell Cycle, Mitotic | Reactome"
 [4] "Cell cycle - yeast - Saccharomyces cerevisiae (budding
yeast) | KEGG"
 [5] "DNA Replication Pre-Initiation | Reactome"
 [6] "DNA Replication | Reactome"
 [7] "DNA replication - Saccharomyces cerevisiae (budding yeast)
| KEGG"
 [8] "DNA strand elongation | Reactome"
 [9] "Establishment of Sister Chromatid Cohesion | Reactome"
[10] "G1/S Transition | Reactome"
[11] "Glycolysis / Gluconeogenesis - Saccharomyces cerevisiae
(budding yeast) | KEGG"
[12] "Lagging Strand Synthesis | Reactome"
[13] "Meiosis - yeast - Saccharomyces cerevisiae (budding yeast)
| KEGG"
```

```
[14] "Mismatch repair - Saccharomyces cerevisiae (budding yeast)
| KEGG"
[15] "Mitotic G1 phase and G1/S transition | Reactome"
[16] "Mitotic Prometaphase | Reactome"
[17] "Oxidative phosphorylation - Saccharomyces cerevisiae
(budding yeast) | KEGG"
[18] "Processive synthesis on the lagging strand | Reactome"
[19] "Removal of the Flap Intermediate | Reactome"
[20] "Resolution of Sister Chromatid Cohesion | Reactome"
[21] "S Phase | Reactome"
[22] "Synthesis of DNA | Reactome"
[23] "Valine, leucine and isoleucine biosynthesis -
Saccharomyces cerevisiae (budding yeast) | KEGG"
[24] "glycolysis III (from glucose) | YeastCyc"
[25] "glycolysis | YeastCyc"

$`LAB:WY23`
[1] "arginine biosynthesis | YeastCyc"

$`WY7:WY15`
 [1] "Activation of the mRNA upon binding of the cap-binding
complex and eIFs, and subsequent binding to 43S | Reactome"
 [2] "Cap-dependent Translation Initiation | Reactome"
 [3] "Eukaryotic Translation Initiation | Reactome"
 [4] "Formation of the ternary complex, and subsequently, the
43S complex | Reactome"
 [5] "L13a-mediated translational silencing of Ceruloplasmin
expression | Reactome"
 [6] "Metabolism of RNA | Reactome"
 [7] "Nonsense Mediated Decay (NMD) enhanced by the Exon
Junction Complex (EJC) | Reactome"
 [8] "Nonsense Mediated Decay (NMD) independent of the Exon
Junction Complex (EJC) | Reactome"
 [9] "Nonsense-Mediated Decay (NMD) | Reactome"
[10] "Ribosomal scanning and start codon recognition | Reactome"
[11] "Ribosome - Saccharomyces cerevisiae (budding yeast) |
KEGG"
[12] "SRP-dependent cotranslational protein targeting to
membrane | Reactome"
[13] "Translation initiation complex formation | Reactome"
[14] "Translation | Reactome"

$`WY7:WY23`
[1] "Lysine biosynthesis - Saccharomyces cerevisiae (budding
yeast) | KEGG"
[2] "superpathway of leucine, valine, and isoleucine
biosynthesis | YeastCyc"
```

```
$`WY15:WY23`
[1] "Citrate cycle (TCA cycle) - Saccharomyces cerevisiae
(budding yeast) | KEGG"
[2] "Citric acid cycle (TCA cycle) | Reactome"
[3] "Pyruvate metabolism and Citric Acid (TCA) cycle | Reactome"
[4] "The citric acid (TCA) cycle and respiratory electron
transport | Reactome"
```

Figure S3. Pathway Enrichment Overlap between *MED15* strains

Raw output for fgsea pathway enrichment analysis corresponding to Venn diagram (Fig. 8). Each grouping corresponds to individual or sets of *MED15* alleles that share the listed enriched pathways from KEGG, Reactome, and YeastCyc databases.

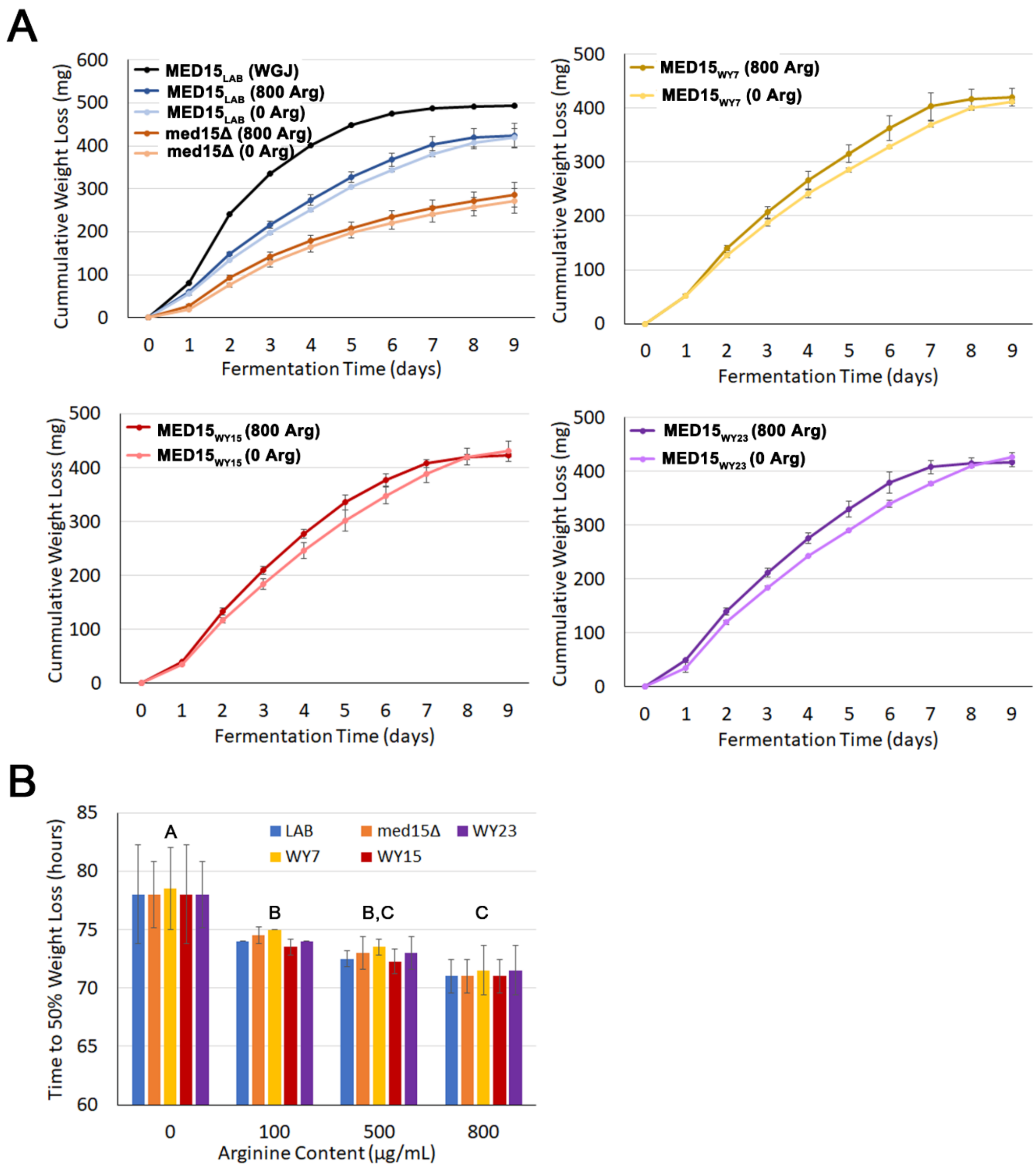

Figure S4. Arginine Supplemented Fermentation

(A) Fermentation curves in SD media (lacking all but strain-essential amino acids) with or without supplementation of arginine across strains with different *MED15* alleles. **(B)** Time to 50% complete fermentation in SD media with different levels of supplementation with arginine across strains with different *MED15* alleles. Significant differences were determined between arginine supplementation groups using ANOVA analysis with a Tukey post-hoc test, Group A – Groups B & C $p<0.001$, Group B – Group C $p<0.01$.

Table S1. Genes with an average of 10,000 or more additional counts using kallisto vs. Bowtie2

Systematic Name	Standard Name	Name Description
YAL005C	SSA1	Stress-Seventy subfamily A
YBL027W	RPL19B	Ribosomal Protein, Large subunit
YBL072C	RPS8A	Ribosomal Protein, Small subunit
YBR010W	HHT1	Histone H Three
YBR031W	RPL4A	Ribosomal Protein, Large subunit
YEL054C	RPL12A	Ribosomal Protein, Large subunit
YKR059W	TIF1	Translation Initiation Factor
YFR031C-A	RPL2A	Ribosomal Protein, Large subunit
YDL191W	RPL35A	Ribosomal Protein, Large subunit
YDR155C	CPR1	Cyclosporin A-sensitive Proline Rotamase
YDR342C	HXT7	HeXose Transporter
YDR345C	HXT3	HeXose Transporter
YDR385W	EFT2	Elongation Factor Two
YDR545W	YRF1-1	
YGR118W	RPS23A	Ribosomal Protein, Small subunit
YGR138C	TPO2	Transporter of POlyamines
YGR192C	TDH3	Triose-phosphate DeHydrogenase
YGR254W	ENO1	ENOlase
YJR145C	RPS4A	Ribosomal Protein, Small subunit
YLR158C	ASP3-3	ASParaginase
YLR466W	YRF1-4	
YML026C	RPS18B	Ribosomal Protein, Small subunit
YML132W	COS3	COnserved Sequence
YMR173W	DDR48	DNA Damage Responsive
YOL086C	ADH1	Alcohol DeHydrogenase
YOR182C	RPS30B	Ribosomal Protein, Small subunit
YPL090C	RPS6A	Ribosomal Protein,Small subunit
YPL220W	RPL1A	Ribosomal Protein, Large subunit
YPR080W	TEF1	Translation Elongation Factor
YDR034C-A	none	
YDR524W-C	none	

Table S2. Genes Differentially Regulated by *MED15*$_{WY23}$ During WGJ Fermentation using Bowtie2-DESeq2 and/or kallisto-DESeq2 methods (log2 fold-change >0.5 or <-0.5 and adjusted p-value <0.05)

**Higher expression in *MED15*$_{WY23}$ strain**		
**Systematic Name**	**Standard Name**	**Name Description**
YBR034C	HMT1	HnRNP MethylTransferase
YDL182W	LYS20	LYSine requiring
YDR011W	SNQ2	Sensitivity to 4-NitroQuinoline-N-oxide
YDR035W	ARO3	AROmatic amino acid requiring
YDR158W	HOM2	HOMoserine requiring
YER062C	GPP2	Glycerol-3-Phosphate Phosphatase
YER069W	ARG5,6	ARGinine requiring
YER073W	ALD5	ALdehyde Dehydrogenase
YER130C	COM2	Cousin of Msn2
YGL009C	LEU1	LEUcine biosynthesis
YGL117W	ARO5	AROmatic amino acid requiring
YGL184C	STR3	Sulfur TRansfer
YGR138C	TPO2	Transporter of POlyamines
YHR018C	ARG4	ARGinine requiring
YHR029C	none	Unknown function
YJL088W	ARG3	ARGinine requiring
YJR109C	CPA2	Carbamyl Phosphate synthetase A
YJR111C	PXP2	PeroXisomal Protein
YKL029C	MAE1	MAlic Enzyme
YKL068W-A	none	Unknown function
YKL120W	OAC1	OxaloAcetate Carrier
YML130C	ERO1	ER Oxidation or Endoplasmic Reticulum Oxidoreductin
YOL058W	ARG1	ARGinine requiring
YOR107W	RGS2	Regulator of heterotrimeric G protein Signaling
YOR202W	HIS3	HIStidine
YOR204W	DED1	Defines Essential Domain
YOR267C	HRK1	Hygromycin Resistance Kinase
YOR302W	CPA1 uORF	Regulates translation of the CPA1 mRNA
YOR303W	CPA1	Carbamyl Phosphate synthetase A
YOR306C	MCH5	MonoCarboxylate transporter Homolog
YOR337W	TEA1	Ty Enhancer Activator

YPL092W	SSU1	Sensitive to SUlfite
**Lower expression in *MED15*$_{WY23}$ strain**		
**Systematic Name**	**Standard Name**	**Name Description**
YDR342C	HXT7	HeXose Transporter
YDR406W	PDR15	Pleiotropic Drug Resistance
YFL054C	AQY3	AQuaporin from Yeast
YKL096W	CWP1	Cell Wall Protein
YKL109W	HAP4	Heme Activator Protein
YMR145C	NDE1	NADH Dehydrogenase, External
YNL194C	none	FMP45 paralog; plasma membrane and eisosome protein
YOR028C	CIN5	Chromosome INstability
YOR161C	PNS1	pH Nine Sensitive
YPL230W	USV1	Up in StarVation
